# Neurodevelopmental benefits of judo training in preschool children: a multinational, mixed methods follow-up study

**DOI:** 10.3389/fpsyg.2024.1457515

**Published:** 2024-12-18

**Authors:** Zuzana Križalkovičová, Péter Szabó, Kata Kumli, Miloš Štefanovský, Alexandra Makai, József Szentpéteri

**Affiliations:** ^1^Department of Sports Sciences, Faculty of Health Sciences, Institute of Physiotherapy and Sport Science, University of Pécs, Pécs, Hungary; ^2^Medical School, Institute of Transdisciplinary Discoveries, University of Pécs, Pécs, Hungary; ^3^Faculty of Humanities, Institute of English Studies, University of Pécs, Pécs, Hungary; ^4^Faculty of Sciences, Institute of Sports Science and Physical Education, University of Pécs, Pécs, Hungary; ^5^National Virology Laboratory, University of Pécs, Pécs, Hungary; ^6^Faculty of Humanities, Institute of Psychology, University of Pécs, Pécs, Hungary; ^7^Laboratory of Combat Sports, Department of Gymnastics, Dance, Fitness, and Combat Sports, Faculty of Physical Education and Sports, Comenius University, Bratislava, Slovakia

**Keywords:** neurodevelopment, judo, motor skills, primitive reflexes, cognitive development, CNS, visual perceptual test

## Abstract

**Introduction:**

In our quasi-experimental study, we evaluated the neurodevelopmental impact of judo on young children (*n* = 182) aged 4–7 years, specifically focusing on primitive reflex integration. Participants were divided into judo and non-judo control groups, and assessments were conducted over 6 months across Hungary, Slovakia, and Austria.

**Methods:**

Neurodevelopmental changes were measured using Institute for Neuro-Physiological Psychology (INPP) and Physical and Neurological Examination for Soft Signs (PANESS) for children, while parents completed the Performance Skills Questionnaire (PSQ).

**Results:**

Analysis with Repeated Measures ANOVA (significance set at *p* < 0.05) revealed significant improvements in cognitive and motor performance in judo-practicing children compared to their non-judo counterparts. Furthermore, Spearman correlation analysis revealed that INPP and PANESS were effective in identifying neurodevelopmental changes, PSQ was not suitable as a simplified screening tool for parents, potentially due to its absence of items focused on primitive reflexes.

**Conclusion:**

Despite the limitations of the study, our findings suggest that judo practice could foster central nervous system (CNS) maturation in young children, promoting the potential inclusion of judo in early childhood education programs.

## 1 Introduction

Primarily, our study explored how judo may promote the inhibition and integration of primitive reflexes, potentially leading to improvements in children’s physical, mental, and cognitive abilities. The aim of our research was to evaluate the beneficial effects of judo on the maturation of the central nervous system in children aged 4–7 years.

### 1.1 Primitive reflexes and CNS maturation

The occurrence of mild brain dysfunction, characterized by motor and coordination disorders as well as cognitive and emotional deficiencies, is unfortunately very much present among children (Biotteau et al., 2020). The most measurable and best-discussed part of these dysfunctions in the literature is Developmental coordination disorder (DCD), a neurodevelopmental condition characterized by a marked impairment in the development of motor skills or motor coordination that develops early-on and interferes with an individual’s activities of daily living (Blank et al., 2019). Prevalence of DCD is estimated to be 5–6% which is most frequently quoted in the literature (Blank et al., 2019; Hua et al., 2022; Downing and Caravolas, 2020) but ranges in reports between 1.4 and 19%, making it one of the more common childhood disorders (Amador-Ruiz et al., 2018) among others like Attention-Deficit/Hyperactivity Disorder (ADHD) and Specific Learning Disabilities (SLD) (Lino and Chieffo, 2022). Children with DCD often appear clumsy and awkward, struggling to integrate themselves into peer activities. By the coming adolescence, most children with motor skill disorders not only perform poorly in physical education but also have a poor physical self-image, and self-regulation and may underperform academically (Elena Žiaková, 2015).

The basis for the theories that are included in developmental kinesiology (DK) is that the development of human motor function in early childhood is genetically pre-determined and follows predictable patterns or programs, which are formed as the central nervous system (CNS) matures, enabling the infant to control posture, achieve erect posture against gravity, and to move purposefully via muscular activity (Frank et al., 2013). From the 25th to the 40th week of gestation in early development, crucial automatic movement patterns called primitive reflexes are formed. Mostly by the sixth month, possibly up to a year, neonatal reflexes ought to be inhibited, to avoid potential problems in development (Chandradasa and Rathnayake, 2020). One of the most respected methods in Europe that deal with reflex integration is the “Vojta” method based on reflex locomotion, which is a neurophysiologic facilitation system for the CNS. Similar to “Vojta” is Dynamic Neuromuscular Stabilization (DNS) a neuromuscular apparatus that consists of components of locomotion: automatic control of posture, uprighting aimed movements (Bauer et al., 1992) in a reciprocal manner. Given a problem with the inhibition of primitive structures, our brain may utilize its property for neuroplasticity to possibly create new synapses and then implications depending on the stimulus strength and frequency of synaptic activation (Chandradasa and Rathnayake, 2020; Hortobagyi et al., 2022; Gulyaeva, 2017; Rahayu et al., 2020; Chan et al., 2022; Melillo et al., 2020).

Evidently, a sedentary lifestyle and retained primitive reflexes can pose a prevalent problem where additional programs and screening may provide helpful data for future endevors (Melillo et al., 2020; Pecuch et al., 2021). In essence, the lack of inhibition might bottleneck the neurodevelopmental processes and cognitive performance (Stephens-Sarlós et al., 2024). Consequently, there is a search for new methods that, alongside classical rehabilitation (therapy improving fine, gross motor skills, and motor planning) (Offor et al., 2016), can help minimize developmental disadvantages (Geuze et al., 2001). Various therapeutic methods promote CNS maturation for improved outcomes, which align well with the effects of sports, particularly judo (Geuze et al., 2001; Yamasaki, 2023; Perrin et al., 2002; Li et al., 2021).

### 1.2 CNS maturation and judo

The relationship between physical activity, psychological wellbeing, and cognition has been discussed in sports science over time (Chaddock et al., 2011; Szabo et al., 2024; Moran, 2009). The aspects of sports are frequency, intensity, duration, and mode of physical activity that provide the greatest synergistic benefit for scholastic achievement and neurocognitive health during childhood (Chaddock et al., 2011). Interestingly, sports, particularly judo might be able to facilitate CNS maturation and help assist in education and therapeutic programs (Geuze et al., 2001; Yamasaki, 2023; Perrin et al., 2002; Li et al., 2021). Judo’s relationship with neurodevelopment and related benefits in adulthood and among the elderly shows promising data (Descamps et al., 2024; Ciaccioni et al., 2024; Harwood-Gross et al., 2021). Furthermore, judo may influence the self-efficacy of practitioners significantly (Moore et al., 2023) during training, competition, and personality development. Judo encompasses the nurturing of courage and confidence, cognitive skills, disciplined behavior, social and physical contact, coordination skills, fall and throw techniques, monotony tolerance, pain tolerance, conditioning, observation skills, and body control (Galla and Horváth, 1982; Bryman, 2006). Judo’s mental training teaches participants how to behave during life’s minor and major upheavals, offering some level of protection from not only physical (Miarka et al., 2020) but also potentially psychological injuries. The mind and thinking must be continuously disciplined to be as resilient as the body through the sport (Gutiérrez García and Pérez Gutiérrez, 2012). Judo movements can be both quantitative and qualitative in nature through their well-defined criteria describing movements that are repeated many times, hence affecting the cardiorespiratory system, and utilizing the body’s nervous system for alignment and strength that applied to new and different positions (Tomporowski et al., 2015). Motor skill interventions that are open-ended, strategic, and sequential in nature are effective in improving cognition (Shi and Feng, 2022). In essence, the goal of judo is to defeat the given opponent with minimal effort. Timing, softness, and perseverance often triumph over significant resistance and force. By effectively training the center of the body, judo athletes increase their ability to generate and maintain power throughout a fight (Barbado et al., 2016). Trunk stability can positively influence performance in a judo match because it facilitates the transfer of forces generated by the lower body to the upper body (and vice versa) during the application of techniques (Kibler et al., 2006). It also improves balance control similar to gymnastics (van Dieen et al., 2012), which is a key factor in coping with the opponents’ disruption of stability (Perrin et al., 2002; Yoshitomi et al., 2006). A stronger core can lead to better coordination of movements and technical skills, resulting in improved overall movement efficiency. It has also been shown that rotatory trunk strength is associated with kinetic variables during pulling movements with a change of position, similar to the Morote-seoi-nage throw (Helm et al., 2020). Maintaining balance while disrupting the opponent’s is crucial during matches ultimately leading to victory.

From another theoretical perspective considering childhood, judo may even resemble rough and tumble play among mammals which is a crucial part of their developmental process, with usually the playfight continuing until one of the participants is on their back on the ground (Blomqvist and Stylin, 2021; Palagi et al., 2016; Pellis et al., 2022). Despite constant movement and positional changes, which naturally involve bodily proprioception, cross-over motions, twists and turns, level change, and overall coordination, efforts must be made to quickly regain lost balance and maintain equilibrium which connects the movements required for the sport with the process of reflex integration (Galla and Horváth, 1982; Chan et al., 2023; Cid-Calfucura et al., 2023; Lubans et al., 2010; Miarka et al., 2020; Miarka et al., 2016). For these reasons, our study aims to explore the connection between CNS maturation in childhood through judo.

In brief, given the longitudinal nature of our study, we hypothesized that children participating in the judo training program would show significant improvement in the second INPP and PANESS measurement due to the sport. Furthermore, we also hypothesized that participants who perform judo exercises would score better on cognitive (visual perceptual test) tests demonstrating neurodevelopment at the second measurement than their non-judo peers. We hypothesized that those who practice judo would perform better on motor tests (neuromotor test) as well, demonstrating neurodevelopment at the second measurement than their non-judo counterparts. Furthermore, we hypothesized that there is a positive correlation between the two tests. Finally, anticipated a significant positive correlation between INPP and PANESS using the Spearman test, to further validate the robustness of our findings. Additionally, if PSQ had shown a significant negative correlation (due to the scoring), we believed it could be a useful tool for parents to screen their children for the relevant issues.

## 2 Materials and methods

### 2.1 Design and sampling

Our quasi-experimental research utilized mixed methods based on quantitative findings from the employed INPP, and PANESS tests integrated (Bryman, 2006) with qualitative results from the PSQ questionnaire. Specifically, INPP and PANESS tests were carried out in person while the questionnaire was distributed using the Google Forms platform to further enhance the data cleaning process where available.

Data collection occurred from January to November 2023 across four countries (Hungary, Slovakia, Croatia, and Austria) in six cities (Pécs, Osijek, Komárno, Gúta, Bratislava, and Vienna). For the Croatian group, we could not carry out repeated measures for our sample, so Croatia was excluded from the statistical analysis. For the map and graphics of study locations and groups (see Supplementary material).

Hungary: Measurements were in Pécs with 101 participants, including two judo groups (*n* = 43) from the “PVSK judo club” and the “Pécsi Sportóvoda” (kindergarten). The control group (*n* = 58) included participants from “Katica Óvoda” (kindergarten) and “Református Óvoda” (kindergarten).

Slovakia: Measurements were with 59 participants in three cities: In Komárno, (*n* = 24) we measured in the “Judo Academy Komárno Judo club” (*n* = 8) and in the Hungarian and Slovak classes in the “Százszorszép Óvoda” (kindergarten) (*n* = 16). In Kollárovo, (*n* = 7) we measured in the “JUDO Klub Gúta.” In Bratislava, (*n* = 28), “Judo Centrum” (*n* = 11) we measured participants starting in a preparatory group (*n* = 3) and participants of more advanced preschool age (*n* = 8). The control group (*n* = 17) was “Waldorfská škôlka Hviezdičky” (kindergarten) (*n* = 7) and “Materská Škôlka Novohorská” (kindergarten) (*n* = 10).

Austria: Measurements were in Vienna with 22 participants from the “WAT Stadlau Judo club” (*n* = 10) and a control group from “Waldorfschulen Hietzing” (kindergarten) (*n* = 12).

Participants were assigned to intervention or comparison groups based on whether they practiced Judo or not and for the duration of their time spent in the sport.

Inclusion criteria: 4–7-year-old participants attending kindergarten and/or regularly training in a judo club. For the purposes of our study based on the literature (Witt, 2018; Crane and Temple, 2014; Fraser-Thomas et al., 2008) participants were eligible to be included in the judo groups if they have been training judo for at least a month. Generally, participants train for 10 months annually (aligned with the school year from September 1 to June 30), twice a week for 1 h (either on non-consecutive days or two consecutive hours in 1 day), totaling approximately 80 h annually.

Exclusion criteria: parental and institutional non-consent, severe orthopedic, cardiovascular, neurological, psychiatric, or endocrine disorders, untreated injuries and trauma, Body Mass Index (BMI) >35 kg/m^2^, lack of motivation, and muscle pain.

The sampling procedure was a non-invasive, purposive cluster sampling technique which we applied for practicality. Initially, we contacted the University of Pécs for ethical approval. Since our study utilizes non-invasive methods of measurement, involving only observation and standard motor skill assessments, we proceeded accordingly. Practice of judo a traditional Japanese martial art, was not the intervention of the study but an already existing variable. Admittedly, we undertook the rigorous process of seeking consent from both the leaders and the parents. Details about the procedure were put in writing to the institutions, and printable information was given to the parents. All parents were informed about the benefits, procedures, and purpose of the study. Willing institutions, namely kindergartens and judo clubs were contacted through formal agreements, ensuring institutional support and cooperation. The selection procedure complied with the Helsinki Declaration principles (World Medical Association Declaration of Helsinki, 2013), ensured voluntary participation without external influence, and anonymized data processing and reporting. We contacted all the accessible kindergartens within the vicinity, and those who consented proceeded to undergo assessment. Clusters of institutions were selected based on their willingness to participate after a telephone conversation, in the cities of our choice due to distance availability. Parents willing to enroll their children in the study gave their consent by signing a Parental Consent Form. Participants were only involved after their consent had been given according to all the compiled consent forms.

Regarding the follow-up measurements, the data collection was carried out by one person with both the first and follow-up measurements. The second measurement has been conducted on the participants, with participation rates varying due to reasons such as illness, transition to school, change of kindergarten, and cessation of sports activity. The general characteristic of the first measurement is observed in Supplementary materials. In our article, only 182 of the remaining participants from the initial 262 are present to keep the article clear and transparent, meaning all the included subjects have been assessed and screened twice for the purposes of robust calculations and statistics. The second measurement was carried out after 6 months, at the same location within the institution. The timing of the pre-and post-measurements was from September 2022 to April 2023 in one school year, the six-month interval was followed for every institution.

After written consent from the institutions, parents were informed by the kindergarten teachers and were able to sign the Parental Consent form and contact us for more information using the contact details provided.

Here it is crucial to consider the key factors for judo training in preschool children (4–7 years) which are the acquisition of movement literacy through the development of locomotion, manipulation, and stabilization skills (Lloyd et al., 2015). In terms of long-term sports training, this stage is referred to as the “Active start” (Balyi, 2012). Judo here serves as an ideal form for the child’s bio-psycho-social development. The training received by the participants in judo clubs lasted 60 min, at a frequency of 2 times per week, and was dominated by general over specific content. Due to the short concentration time, the children acquired the specific coordination skills of judo in the first part of the training, right after the dynamic warm-up. These included falling techniques, immobilization, and throwing techniques in an adapted form for this age category. The main part of the training consisted of structured play. In particular, chases, relays, obstacle courses, and simple fighting games were used, which primarily develop strength and speed, core stability, cognitive functions, courage, and fair play. The end of the training was directed toward calming the body, through simple stretching exercises, passive lying rest with eyes closed or quizzes oriented to Japanese judo terminology.

However, participants were not trained for the study’s measurements, and they were not prepared for the screening exercises. The target group of the research included participants for whom the head of the preschool institution or the judo club agreed to the measurement in the institution’s building and allowed the participants to leave the group for the duration of the survey, interrupting the session for about 20 min in the presence of an assistant teacher/coach. If the participant was not outright excluded from the sampling the measurements began. The room used for the study had to be of an appropriate size with adequate lighting, minimal noise, and as little extraneous material as possible to avoid unnecessary physiological stress and potential distractions. Other necessary items were a stopwatch, a chair for the examiner, a chair for the child facing the examiner, and a table. It was crucial to find a place on the floor to guide the participants along the two-meter line, if this was not possible, we had to place a colored 1 cm wide adhesive tape on the floor away from surrounding objects.

The tests were performed with the participants wearing loose clothing and walking barefoot. This neurological examination was designed to help determine the presence of subtle neurological symptoms. The tests did not assess the participant’s learning abilities at that time, meaning it was important that the participant fully understood through verbal guidance what was expected from them, with all the tasks to be completed as described.

Consequently, a positive atmosphere was maintained through verbal praise and positive reinforcement. Gentle verbal correction was used when the child did not understand the task. The interaction with each participant had to be done in a few minutes so as not to lose the participants’ attention. In order to compensate for the attention problem, the two test’s items were woven into each other, resulting in participants not perceiving how long one or the other set of exercises took. Participants always began with simple exercises, with static stances, and then increased difficult exercises.

### 2.2 Instruments

The INPP test is recognized as a reliable test among practitioners focused on neurodevelopmental issues, educational psychology, and occupational therapy (Blythe, 2005). It is generally practical for assessing certain aspects of neurological and motor development in children. It is based on established theories of neurophysiology and has been used in various studies to examine developmental delays and neurodevelopmental disorders (Blythe, 2005; Blythe, 2014). The INPP test assessed postural reflex actions, crucial for efficient cognitive functioning (Pecuch et al., 2021). In our study the calculated reliability measurement of the INPP test involving Cronbach’s alpha and McDonald’s omega were (0.793) and (0.812), in the first measurement and in the second (0.797) and (0.826) respectively (see Supplementary material).

INPP includes the Romberg test as well, which is used to assess proprioception and examine static balance (Blythe and Hyland, 1998). The single-leg test is the ability to control static balance and equilibrium by using one side of the body independently of the other. In addition to maintaining balance while standing on one leg, Schrager’s test (De Quiros and Schrager, 1979) has shown that observing timing and body position during the test while standing on one leg can provide additional information about the maturity as well as the development of the CNS (Blythe, 2014). Regarding the asymmetric tonic neck reflex (ATNR), turning the head to one side causes the arm and leg to extend on the same side, and on the opposite side, the child retracts the arm and leg (Parmenter, 1983). The symmetrical tonic neck reflex (STNR) in preschool-age participants can be elicited in a quadrupedal position. Considering the STNR the head is extended, the tone of the extensor muscles in the arms increases, and the tone of the flexor muscles decreases (in the hips and knees). When the head is anteflexed, the flexor tone of the arms increases, alongside the tone of the hip and knee muscles (Blythe, 2017a). The tonic labyrinth reflex (TLR) is a primitive response to gravity that regresses with the development of head control, muscle tone, and postural control (Blythe, 2017b). Tansley standard Figures are based on drawing tests originally developed by Gesell to assess fine motor skills and visual-perceptual motor skills (Blythe, 2005).

The test examined balance, proprioception, and coordination, and included static balance and the ability to perform tasks involving crossing the body midline. Balance control not only provides physical stability of movement in space but is also a key reference point for cognitive operations in space, including orientation, direction perception, and understanding of spatial mental operations such as addition and subtraction, multiplication, and division (Blythe, 2005). Furthermore, the test consists of two subtests, one testing the participant’s cognitive abilities (visual perceptual test) and the other their motor skills (neuromotor test). The maximum score for the visual perceptual test was 24 points, the maximum score for the neuromotor test was 60 points and the maximum score for the INPP test was 96 points. The lower the participant’s scores on the test, the better their performance. Score ranges regarding INPP were determined as follows: 0 meant No abnormality detected; 1 reflex present to 25 or 25% dysfunction in carrying out the task successfully; 2 reflexes present to 50 or 50% dysfunction in carrying out the task successfully; 3 reflexes present to 75 or 75% dysfunction in carrying out the task successfully; 4 reflexes retained (100%) or unable to carry out the task successfully.

The Physical and Neurological Examination for Soft Signs (PANESS) test was validated and revised by Martha Denckla (Denckla, 1985). It is an internationally accepted valid measurement tool for health professionals and neurologists (Gidley Larson et al., 2007). All the examiner needs are a stopwatch and a score sheet, and it takes only about 30 min. Moderate to excellent inter-rater reliability was identified across PANESS subscores and total scores. The strongest inter-rater reliability was observed for the Timed Motor portion of the PANESS (ICCs >0.90) (Svingos et al., 2023). The PANESS has adequate test–retest and inter-rater reliability (kappa ≥0.5; intraclass coefficient ≥ 0.7), internal consistency (Cronbach’s alpha = 0.74), and sensitivity to age-related changes (Cole et al., 2008; Stephens et al., 2018; Vitiello et al., 1989; Crasta et al., 2021). In our study the calculated reliability measurement of the PANESS test involving Cronbach’s alpha and McDonald’s omega were (0.814) and (0.862), in the first measurement and in the second (0.823) and (0.875) respectively (see Supplementary material). PANESS includes two subscores—Gaits and Stations, and Total Timed—which are summed to produce a Total score (136 points maximum); higher values indicate poorer performance. The Gaits and Stations subscore assesses balance and walking disturbances, as well as excessive motor movements and irregular posture or muscle tone during task execution. The Total Timed score evaluates speed and accuracy deficits in repetitive and patterned motor tasks, along with irregularities in rhythm and overflow of movements (Stephens et al., 2018). The test variables are lateral preference, gait, balance, motor endurance, coordination, and overflow (motor “overflow” refers to the combined movement of body parts not specifically required to perform a task effectively). There are several different forms of motor overflow: associated movement, contralateral motor irradiation and mirror movement (Pitzianti et al., 2017), rhythmic movement disorder, and timed movements (repetitive and patterned) (Dickstein et al., 2005).

The Performance Skills Questionnaire (PSQ) is a validated performance skills questionnaire containing 34 items in three areas: motor skills (10 items), process skills (14 items), and communication skills (10 items). Each item was scored by parents on a Likert scale of 1 to 6, with a higher score indicating better performance skills (maximum score is 204). Parents were asked to rate the extent to which each item characterized their child (very characterized my child - not at all characterized my child). The PSQ yielded three measures, one for each domain, and an overall score (Stephens et al., 2018). Cronbach’s coefficient alpha of the whole sample for motor skills, process skills, and communication skills was (0.89, 0.92, and 0.84) respectively in another study (Bart et al., 2010). In our study the calculated reliability measurement of the PSQ test involving Cronbach’s alpha and McDonald’s omega were (0.954) and (0.959) respectively (see Supplementary material). Data collection occurred after the first measurement. Parents who signed the consent form and were interested in receiving their children’s results by email were sent the questionnaire. Questionnaires were distributed using the Google Forms platform from September to November 2022. Parents of 50 Hungarian, 49 Slovak, and 8 Austrian participants completed the Supplementary questions about pregnancy, delivery, and health of participants and the PSQ (Performance Skills Questionnaire) (see Supplementary materials), totaling 107 questionnaires, with a 40.84% response rate. The low response rate was due to the fact that only parents who were interested in receiving their child’s test results and willing to complete the questionnaire on the online platform filled it out. The measurement was conducted not only to provide a snapshot of the participants but also to assess how the participants were performing by incorporating a third party, typically a parent who spends daily activities with their children. For statistical purposes, only 80 out of the 107 responses were utilized, due to the fact that some of the children were not measured in the second sampling.

### 2.3 Statistical analysis

For statistics, descriptive statistics, and the illustration we utilized Jamovi (ŞAhİN and Aybek, 2020) (Version: 2.6.13) and Power BI (Becker and Gould, 2019) (Version: 2.137.1102.0) (see Supplementary materials). Statistical data collection and processing involved descriptive statistics (mean, standard deviation) and normality tests. To test the association between the measurement tools correlation analyses (Spearman variation) were used.

The sample size required was calculated based on a possible loss of 20% for data analysis to detect between-group differences, with an estimated effect size of 0.50, and a significance level adopted as 0.05, statistical power of 0.80, the minimum number of children for each group (judo and kindergarten) was 61. G*Power software (Kang, 2021) (version 3.1.9.7; Heinrich-Heine-Universität Düsseldorf, Düsseldorf, Germany) was used for estimating the minimum number of the individuals participating in the research. To examine the effect of the intervention ANOVA analysis was performed where the first measurement’s results were used as covariates.

First, we applied Repeated Measures ANOVA tests to see whether there was a significant difference between the improvement of judo-practicing and non-judo individuals between the first and second time of measurement on the INPP and PANESS tests. For the *post-hoc* analyses, we used Tukey correction. Then, we ran Mann–Whitney *U* tests to observe the potential difference between judo and non-judo participants in the second visual perceptual test and the second neuromotor test. In addition, we used One-way ANOVAs with Games-Howell *post hoc* tests to reveal whether there was a difference between participants having practiced judo for different time periods and participants with no judo experience.

## 3 Results

The samples comprised 105 non-judo control group participants (57.69%), 44 participants with <1 year of judo practice (24.18%), 27 participants with more than 1 year of judo practice (14.84%), and 6 participants with more than 2 years of practice (3.29%). The sample included 116 males (64.44%) and 66 females (36.67%), with an average age of 4.92 years. Results of the first measurement, the correlation matrix and descriptive statistics are available in the Supplementary material.

**Figure 1:**
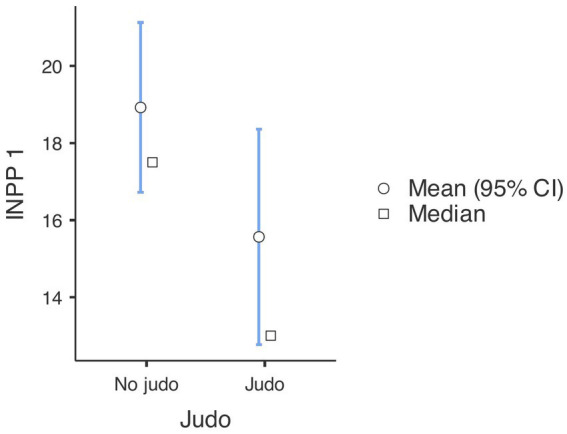
INPP scale test results at the first measurement (*n* = 182). Lower values represent a better performance.

**Figure 2:**
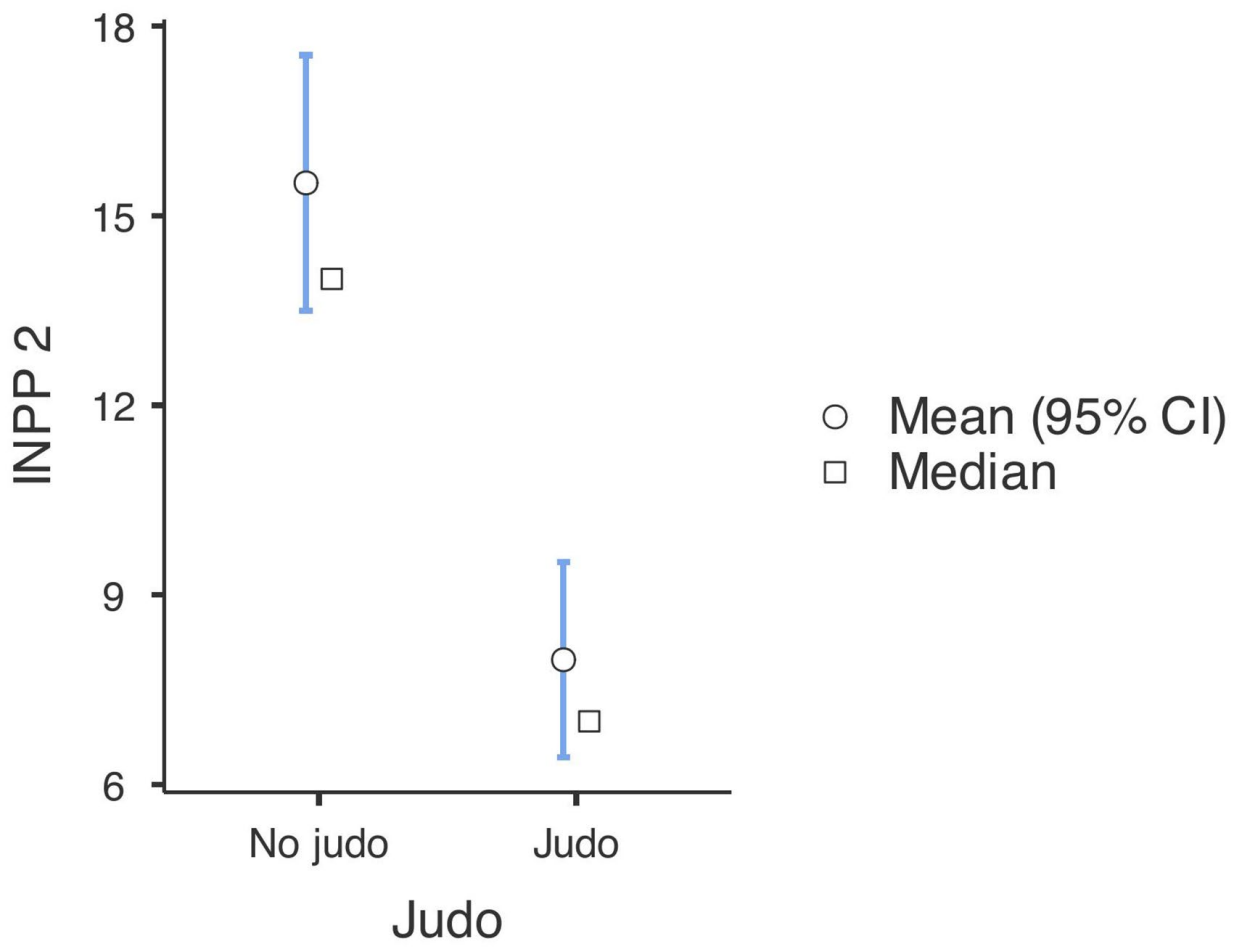
INPP scale test results at the second measurement (*n* = 182). Lower values represent a better performance.

First, a Repeated Measures ANOVA revealed a significant difference in the INPP results of both non-judo and judo-practicing participants between the first and second time of measurement, indicating improvement [*F*_(1, 180)_ = 15.51, $\eta_{G}^{2}$ = 0.005, *η*^2^ = 0.003, $\eta_{p}^{2}$ = 0.079, *p* < 0.001] (see Figure 1, 2). *Post hoc* analyses with Tukey correction showed that in the case of judo-practicing participants (first measurement: M = 15.56, SE = 1.355; second measurement: M = 7.97, SE = 1.037), the difference between the first and second measurements was bigger [*t*(180) = 8.78, *p* < 0.001] than in the case of non-judo participants (first measurement: M = 18.92, SE = 1.173; second measurement: M = 15.52, SE = 0.898) [*t*(180) = 4.55, *p* < 0.001]. This means that subjects doing judo made more improvement between the two measurements than non-judo participants.

**Figure 3:**
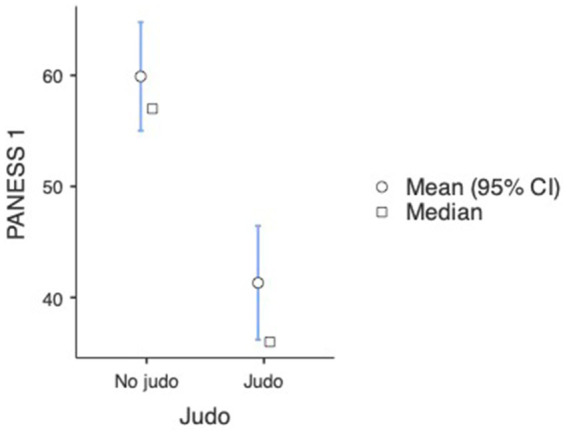
PANESS scale test results at the first measurement (*n* = 182). Lower values represent a better performance.

**Figure 4:**
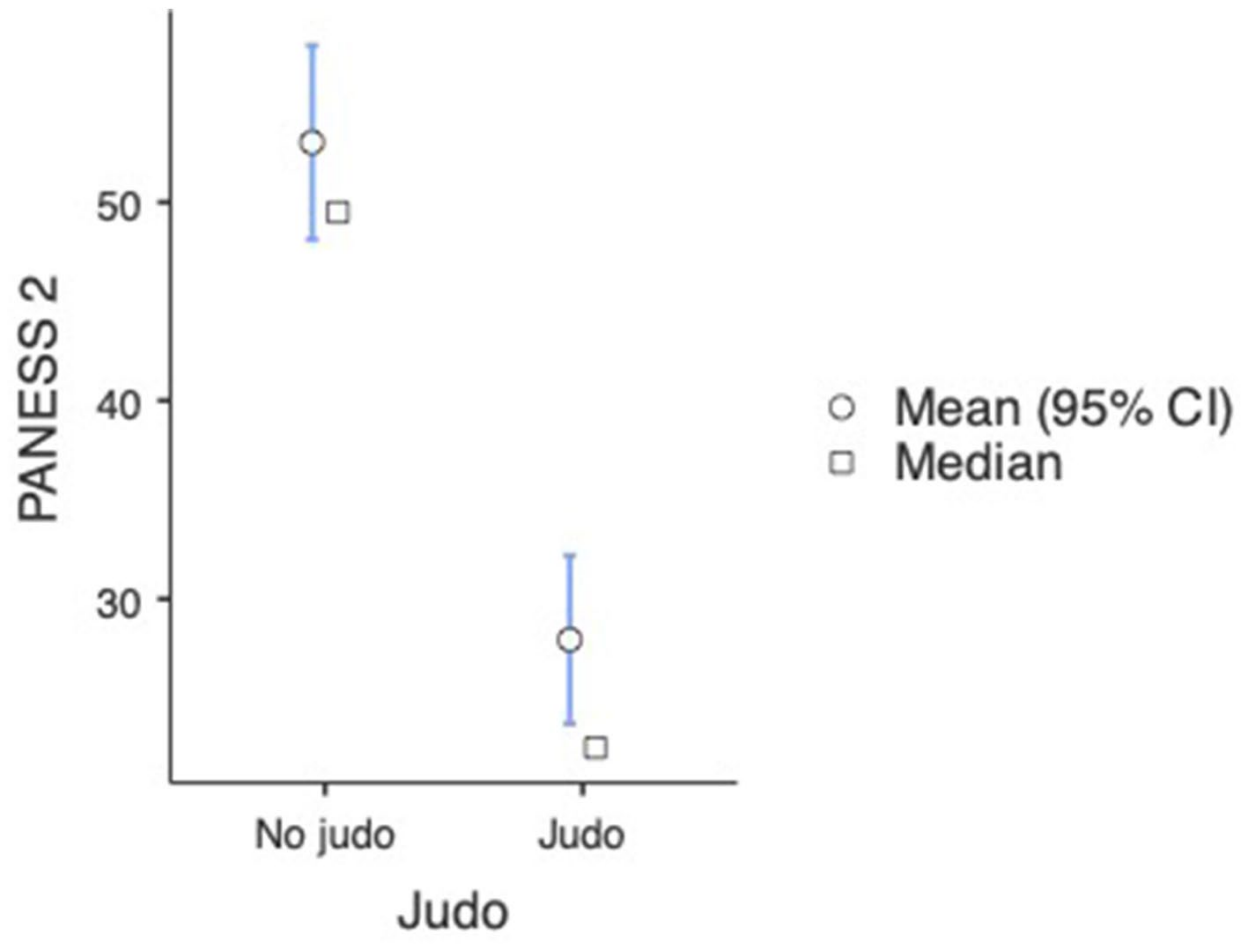
PANESS scale test results at the second measurement (*n* = 182). Lower values represent a better performance.

**Figure 5:**
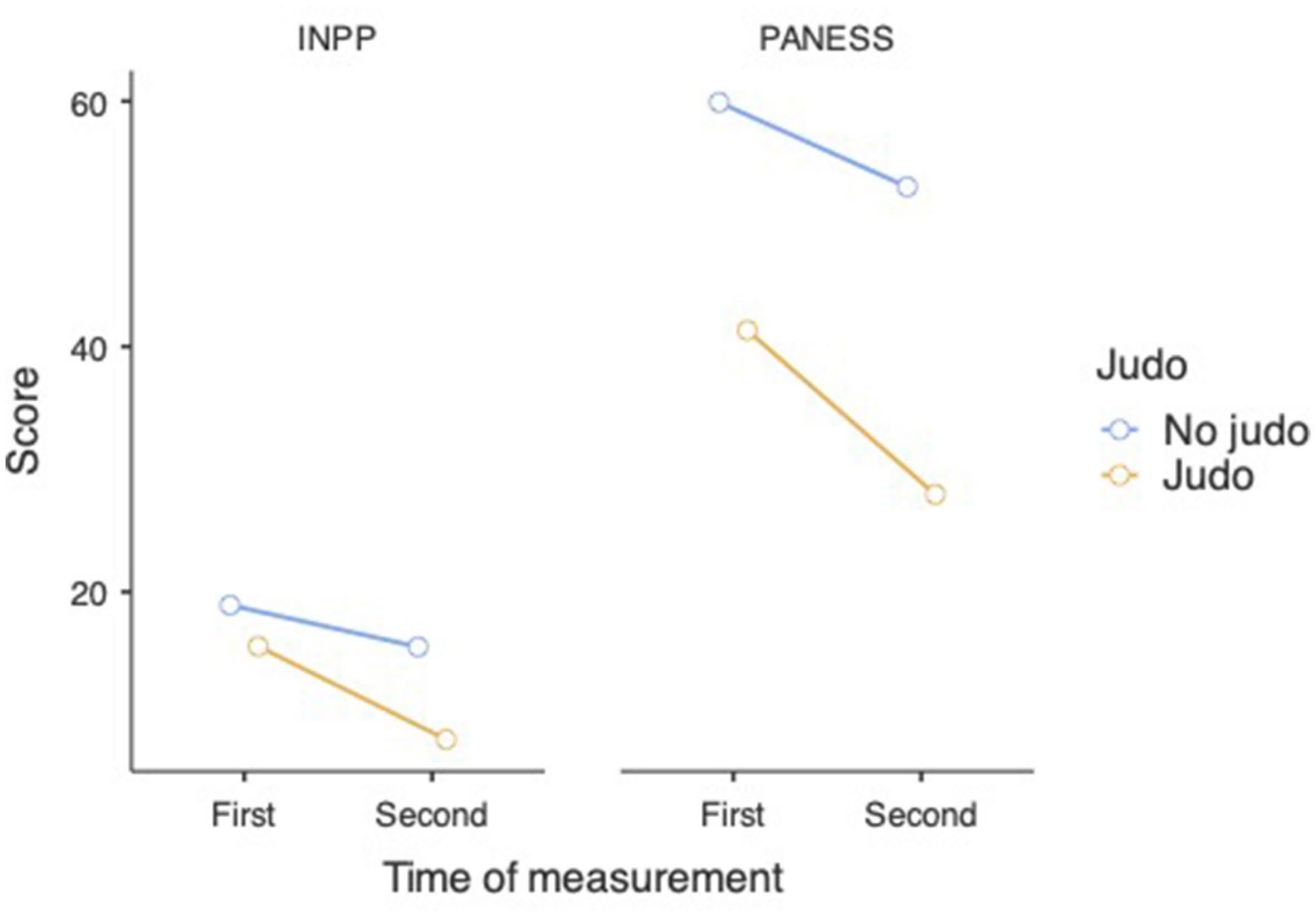
Results of tests demonstrating development in the pre-post measurements (*n* = 182). Lower values represent a better performance.

The repeated measures ANOVA also showed a significant difference in the PANESS results of both non-judo and judo-practicing participants between the first and second time of measurement, indicating improvement here as well [*F*_(1, 180)_ = 15.51, $\eta_{G}^{2}$ = 0.005, *η*^2^ = 0.003, $\eta_{p}^{2}$ = 0.079, *p* < 0.001] (see Figures 3, 4). Just like in the INPP measurement, *post hoc* analyses with Tukey correction showed that in the case of judo-practicing participants (first measurement: M = 41.32, SE = 2.770; second measurement: M = 27.95, SE = 2.599), the difference between the first and second measurements was bigger [*t*(180) = 9.16, *p* < 0.001] than in the case of non-judo participants (first measurement: M = 59.90, SE = 2.399; second measurement: M = 53.02, SE = 2.250) [*t*(180) = −3.28, *p* = 0.027] (see Figure 5 and Table 1). This indicates that participants practicing judo made more improvement between the two measurements than participants with no judo experience.

**Table 1:** Means, standard deviations for outcomes in INPP and PANESS measures (*n* = 182).

Group descriptives						
	Group	*N*	Mean	Median	SD	SE
INPP 1	No judo	104	18.9	17.5	11.5	1.13
	Judo	78	15.56	13.00	12.60	1.426
INPP 2	No judo	104	15.5	14.0	10.5	1.03
	Judo	78	7.97	7.00	6.94	0.786
PANESS 1	No judo	104	59.9	57.0	25.4	2.49
	Judo	78	41.32	36.00	23.09	2.615
PANESS 2	No judo	104	53.0	49.5	25.4	2.49
	Judo	78	27.95	22.50	19.12	2.165

**Figure 6:**
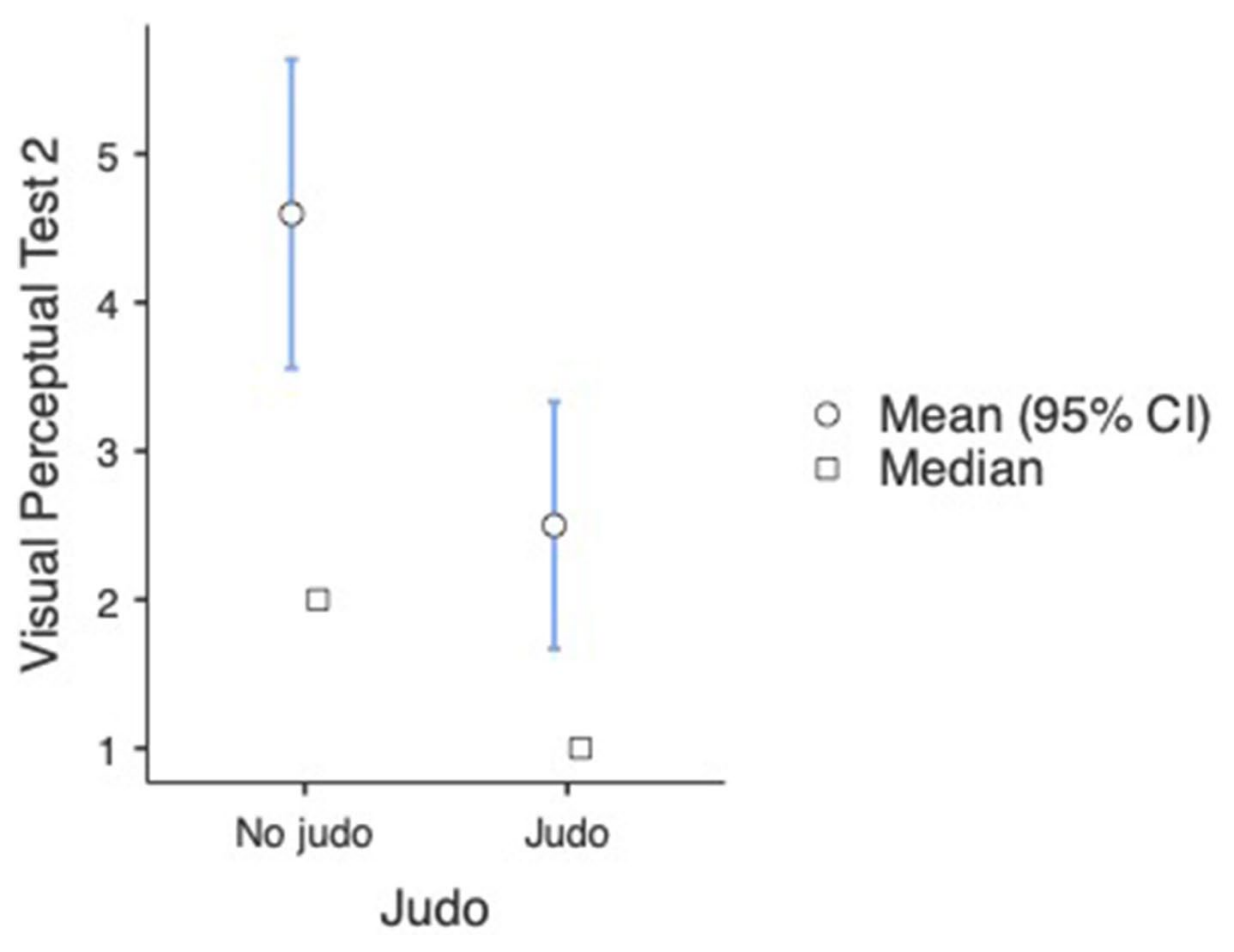
Visual perceptual test results at the second measurement (*n* = 182). Lower values represent a better performance.

**Figure 7:**
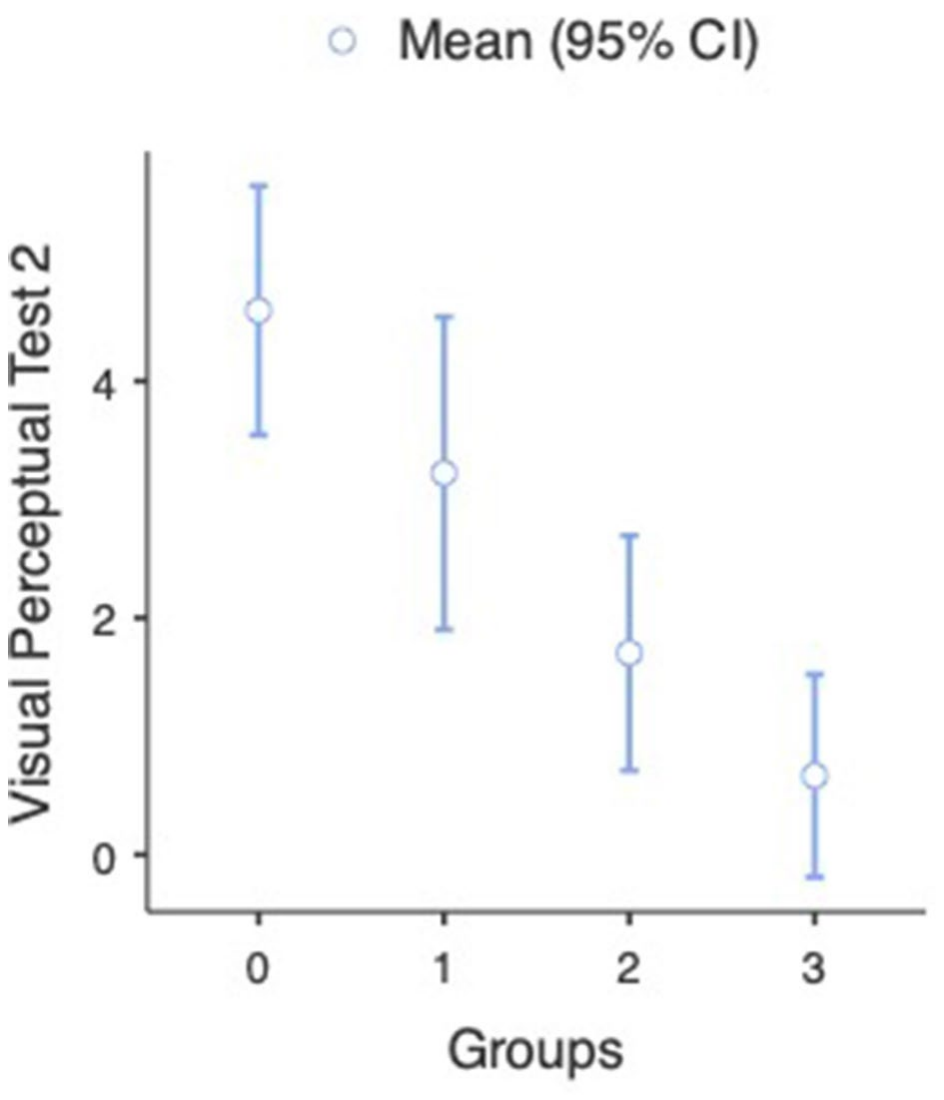
Visual perceptual test results at the second measurement (*n* = 182). 0-non-judo participants, 1-participants with <1 year of judo experience, 2-participants with more than 2 year of judo experience, and 3-participants with more than 2 years of judo experience. Lower values represent a better performance.

Regarding the difference between participants participating in the judo training program and their non-judo peers in the second measurement, the results were the following: Mann–Whitney U tests indicated that a significant difference can be found between participants having practiced judo for different time periods and participants with no judo experience in the second visual perceptual test (U = 3,148, *p* = 0.009). The analysis revealed that the subjects’ visual perceptual test score was significantly lower (better) in the case of judo-practicing participants (M = 2.50, SD = 3.74, SE = 0.424) than participants without judo experience (M = 4.60, SD = 5.41, SE = 0.531) (see Figure 6). We used the non-parametric alternative of the independent samples t-test as the assumption of normality was violated (Shapiro–Wilk *p* < 0.001). A One-way ANOVA revealed that a significant difference was found between participants with different amounts of judo training [*F*_(3, 48.3)_ =14.2, *p* < 0.001]. Games-Howell *post hoc* tests showed that compared to non-judo participants (M = 4.60, SD = 5.41, SE = 0.531), participants with more than 1 year of judo experience (M = 1.70, SD = 2.51, SE = 0.483) [*t*(92.6) = 4.03, *p* < 0.001] and participants with more than 2 years of experience (M = 0.67, SD = 0.82, SE = 0.333) (t(47.6) = 6.27, p < 0.001) got significantly better results on the second visual perceptual test. Between non-judo participants and participants with <1 year of judo experience (M = 3.22, SD = 4.40, SE = 0.656), there was no significant difference [*t*(102) =1.63, *p* = 0.367] (see Figure 7).

**Figure 8:**
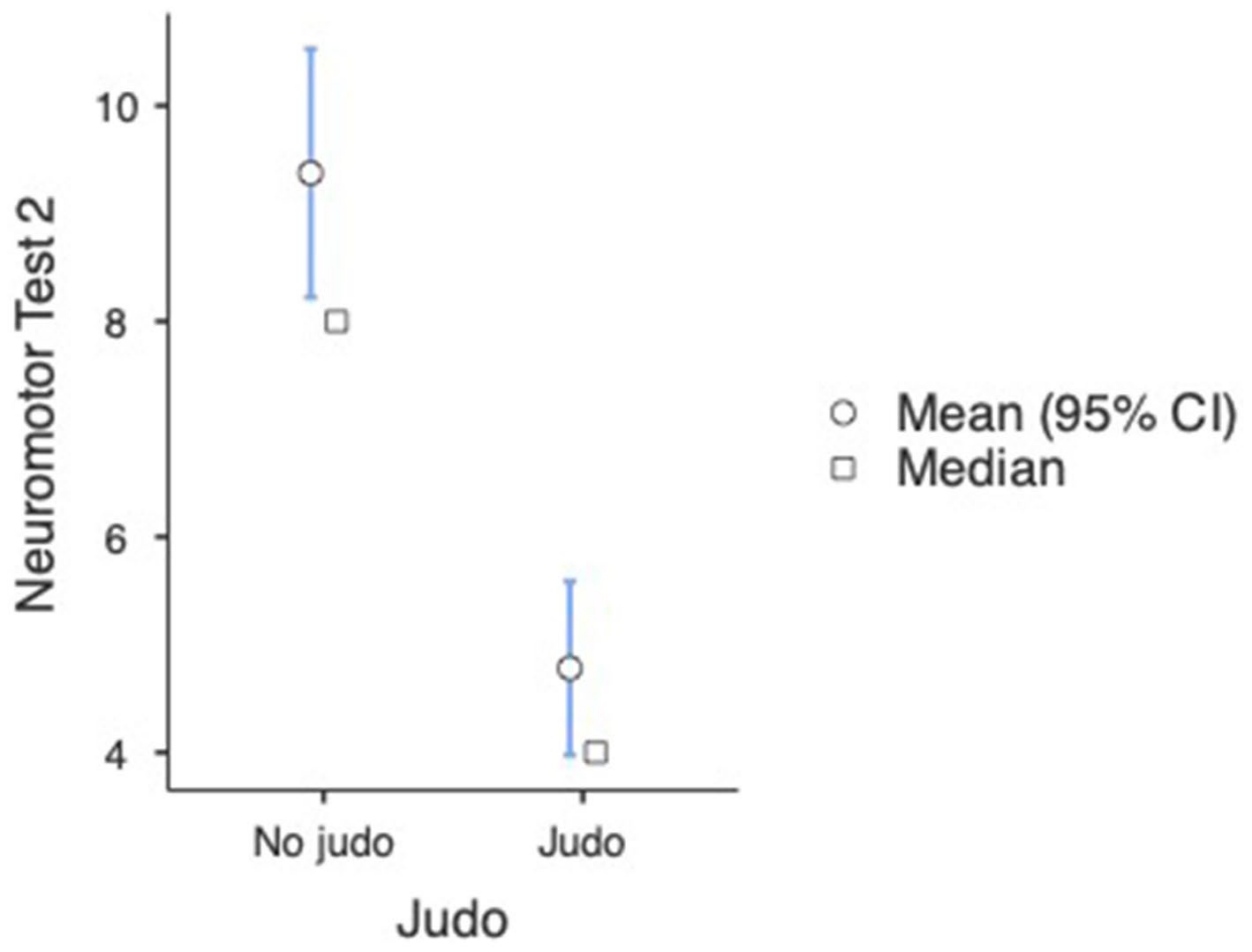
Neuromotor test results at the second measurement (*n* = 182). Lower values represent a better performance.

**Figure 9:**
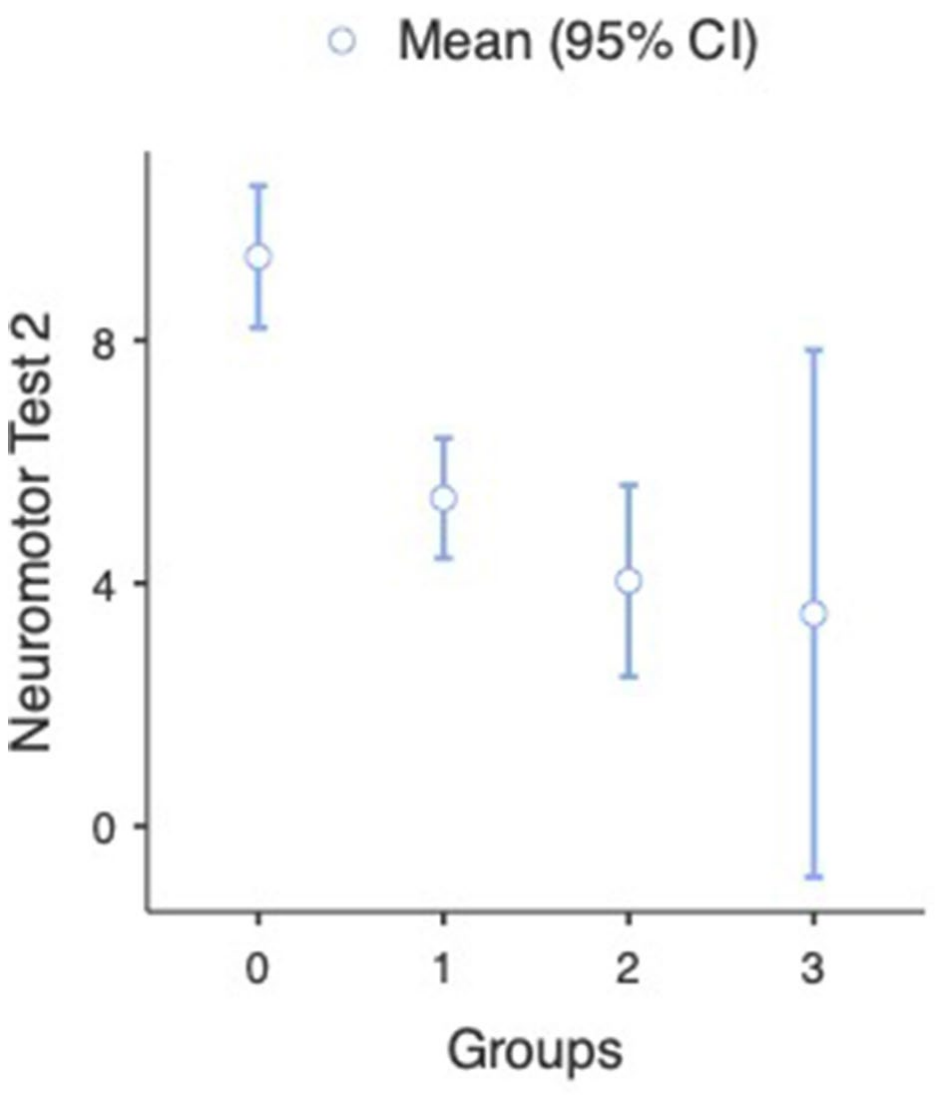
Neuromotor test results at the second measurement (*n* = 182). 0-non-judo participants, 1-participants with <1 year of judo experience, 2-participants with more than 1 year of judo experience, and 3-participants with more than 2 years of judo experience. Lower values represent a better performance.

In the case of the second neuromotor test, we also found a significant difference between judo practitioners and non-judo subjects (U = 1969, *p* < 0.001). The analysis indicated that the judo subjects’ neuromotor test scores were significantly lower (better) in the case of participants doing judo (M = 4.78, SD = 3.63, SE = 0.412) than subjects without judo experience (M = 9.38, SD = 6.00, SE = 0.588) (see Figure 8 and Table 2). A One-way ANOVA revealed that a significant difference was found between participants with different amounts of judo training [*F*_(3, 22.6)_ = 13.3, *p* < 0.001] (see Figure 9 and Table 3). Games-Howell *post hoc* tests showed that compared to non-judo participants (M = 9.38, SD = 5.60, SE = 0.588), participants with <1 year of experience (M = 5.40, SD = 3.29, SE = 0.491) [*t*(139) = 5.19, *p* < 0.001] and participants with more than 1 year of experience (M = 4.04, SD = 3.99, SE = 0.767) [*t*(60.3) = 5.52, *p* < 0.001] got significantly better results on the second neuromotor test. Between non-judo participants and participants with more than 2 years of judo experience (M = 3.50, SD = 4.14, SE = 1.688), there was no significant difference [*t*(6.28) =3.286, *p* = 0.058].

**Table 2:** Group descriptives at the second visual perceptual and neuromotor tests measurement (*n* = 182).

Group descriptives						
	Group	*N*	Mean	Median	SD	SE
Neuromotor test 2	No judo	104	9.38	8.00	6.00	0.588
	Judo	78	4.78	4.00	3.63	0.412
Visual perceptual test 2	No judo	104	4.60	2.00	5.41	0.531
	Judo	78	2.50	1.00	3.74	0.424

**Table 3:** Group descriptives at the second visual perceptual and neuromotor tests measurement (*n* = 182).

Group descriptives					
	Groups	*N*	Mean	SD	SE
Neuromotor test 2	0	104	9.375	5.995	0.588
	1	45	5.400	3.292	0.491
	2	27	4.037	3.985	0.767
	3	6	3.500	4.135	1.688
Visual perceptual test 2	0	104	4.596	5.410	0.531
	1	45	3.222	4.400	0.656
	2	27	1.704	2.509	0.483
	3	6	0.667	0.816	0.333

0-non-judo participants, 1-participants with <1 year of judo experience, 2-participants with more than 1 year of judo experience, and 3-participants with more than 2 years of judo experience.

Finally, regarding the connection between our measurement tools, we observed a strong positive correlation (Spearman correlation analysis) between the first INPP and first PANESS test results [rho (78) = 0.641, *p* < 0.001] and also the second INPP and second PANESS test results [rho (78) = 0.777, *p* < 0.001], further proving the reliability of our testing method. However, neither the first (*p* = 0.052) nor the second INPP test results (*p* = 0.055) correlated significantly with the PSQ questionnaire. Moreover, neither the first (*p* = 0.379) nor the second PANESS test results (*p* = 0.605) correlated significantly with the PSQ questionnaire (see Supplementary material).

## 4 Discussion

The aim of our study was to explore how judo training could influence the maturation and inhibition, or integration of primitive reflexes related to improvement in physical, mental, and cognitive abilities among children between 4 and 7 years through the INPP and PANESS tests. Our results are consistent with our hypotheses: participation in judo training increased neurodevelopmental variables compared to non-judo peers.

In mammals the rough and tumble play with parents and among peers reflects similar characteristics to combat sports (Pellis and Pellis, 2007; Blomqvist and Stylin, 2021; Palagi et al., 2016; Fletcher et al., 2011), particularly judo, because the fight lasts until one party is defeated and is on the ground, unable to move. Most likely this is to prepare for the required movements for survival through the practice fight reflecting on the topic at hand.

First, both judo and non-judo groups showed improvements in INPP and PANESS over the 6 months, although, these changes likely reflect the expected developmental progression in this age group (Crasta et al., 2021; Infante-Cañete et al., 2023; Goddard Blythe et al., 2021; Sweeney et al., 2018). However, given the advantages of physical activity in childhood, the judo group showed more improvements from the first to second measurement, which may suggest an acceleration in CNS maturation with judo as a practically suitable physical activity (Pecuch et al., 2021; Infante-Cañete et al., 2023; Goddard Blythe et al., 2021; Gomes da Silva and Arida, 2015; Jing et al., 2024; Liu et al., 2024). Interestingly, participants with more than 1 year of judo experience scored better on cognitive (visual perceptual test) and motor (neuromotor test) measurements compared to non-judo participants.

Meanwhile, a significant positive correlation was identified between INPP total test batteries and PANESS supporting the validity and robustness of both of our measurements and findings. The INPP and PANESS assessments reinforce each other as complementary tools for evaluating neurodevelopmental progress, reinforcing their utility in both clinical and research settings.

Regarding the search for screening methods and potential solutions, the PSQ questionnaire is not a suitable solution for parents to screen their children for specific issues regarding the neurodevelopmental variables that judo can help with. PSQ lacks the sensitivity to capture specific improvements in primitive reflex integration observed by the two other tests. This highlights the need for objective, performance-based measures when assessing neurodevelopmental outcomes in children that are easily accessible to parents.

Consequently, our findings have important implications for early childhood education and intervention programs. Incorporating activities like judo into preschool curricula may enhance neurodevelopmental outcomes, particularly for children at risk of developmental delays. Early intervention is crucial, as the brain is highly plastic during this period, and appropriate stimulation can lead to significant long-term benefits.

Future research could consider randomized controlled trials to strengthen the causal inferences indicated in this study. Investigating the specific elements of judo training that contribute most to neurodevelopmental gains could inform the design of targeted interventions like neuroimaging and detailed analysis of judo movements for primitive reflex integration exercises. Additionally, exploring the long-term effects of sustained judo practice on academic achievement and psychosocial wellbeing would provide valuable insights for educational systems.

Our findings about CNS maturation and judo’s role in balance, proprioception, and sensorimotor adaptations align well with other studies in this area, however further investigation is required on the direct impact of martial arts and judo on cognitive development.

### 4.1 Limitations

Regarding the limitations of our study, we observed the following. Since quasi-experimental designs do not use random assignment, selection bias may occur here, which may affect the internal validity of our results. Our sample may have been influenced by the fact that groups of institutions were selected based on their willingness to participate, but we tried to compensate for this by measuring in more countries and more cities. Second, without randomization, it is challenging to control for external variables that may influence the results, potentially leading to spurious relationships, like parents may not have signed the consent form because they feared their child would do badly on the tests. The kindergartens that contacted us could have been institutions that took better care of their children’s development and wanted to prove to themselves and their parents that they were doing well. The results may also have been influenced by the fact that children who have been practicing judo for longer periods were either allowed to train for longer periods because they are exceptionally talented or because they are developmentally delayed and utilize more training to improve their condition. Third, the drop-out rate of children in the second measurement was high. Some of them changed kindergartens and judo clubs or quit the sport. This resulted in a significant drop in the number of items returned, which may limit the generalizability of the results. Fourth, we recorded data at two points in time, limiting the ability to infer causal relationships or changes over time, and in the age group 4–7 years, participants could lose attention and motivation relatively quickly, as behavior and cooperation could not be constant (Pellis and Pellis, 2007; Blomqvist and Stylin, 2021; Palagi et al., 2016; Fletcher et al., 2011).

## 5 Conclusion

In this study, the neurodevelopment of preschool children who practiced judo was significantly better than that of their non-judo preschool peers. In summary, we found that judo and its preparatory exercises are beneficial, as they encourage natural movement patterns and proper body control, which is crucial for effective motor performance. A further avenue of research shall involve the meticulous biomechanical analysis of judo movements and their effects on the central nervous system using an object-based imaging method for a better understanding of the judo’s effect on the CNS while taking the primitive reflexes into account.

## Data Availability

The raw data supporting the conclusions of this article will be made available by the authors, without undue reservation.
