# Supplementary material for: Neurodevelopmental benefits of judo training in preschool children: a multinational, mixed methods follow-up study
Source: Front Psychol. 2024 Dec 18;15:1457515. doi: 10.3389/fpsyg.2024.1457515 (PMC11691969; doi:10.3389/fpsyg.2024.1457515)
Supplement: Supplementary file 1 [file Data_Sheet_1.PDF]

Gender Distribution (n=182)

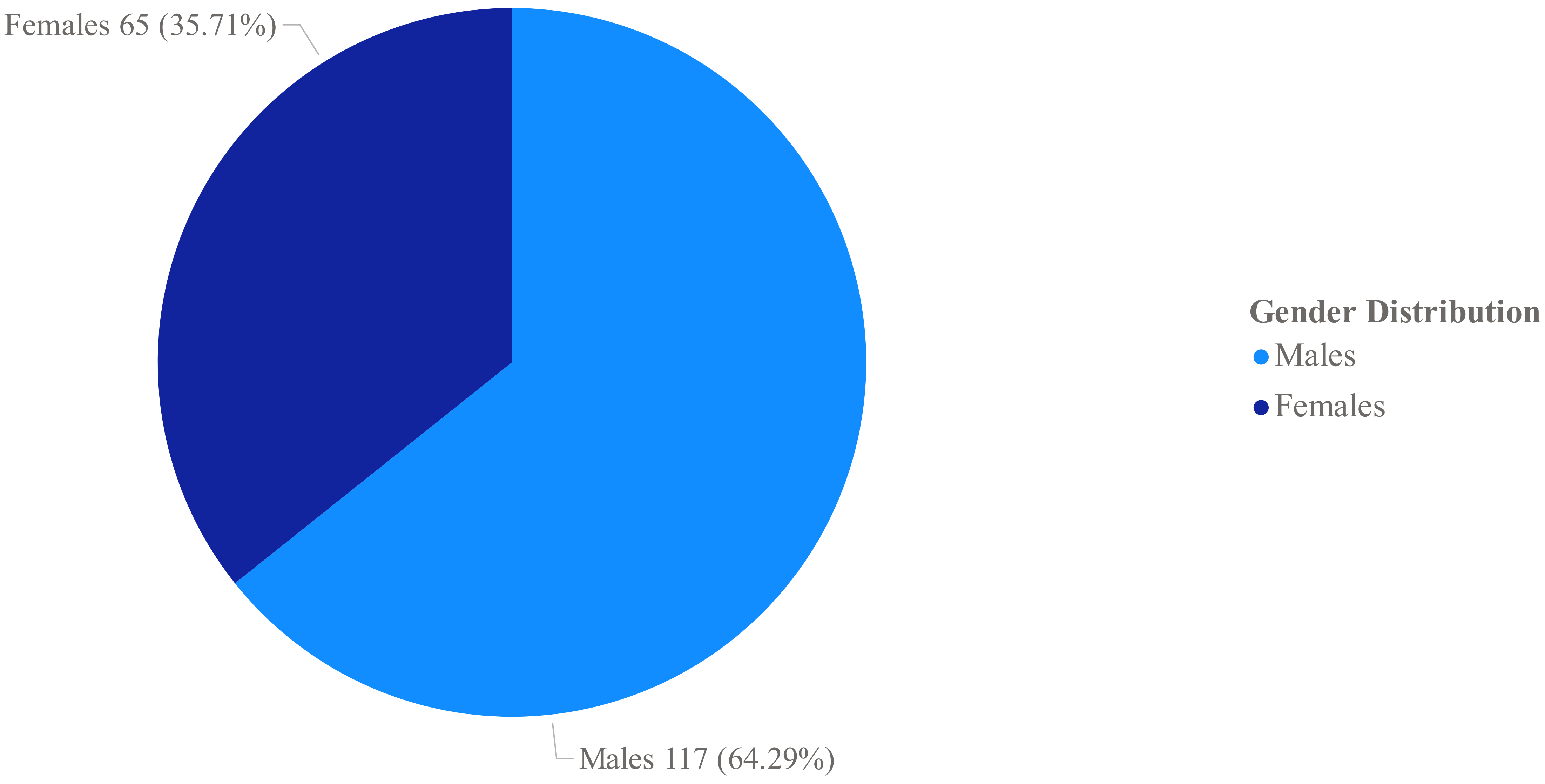

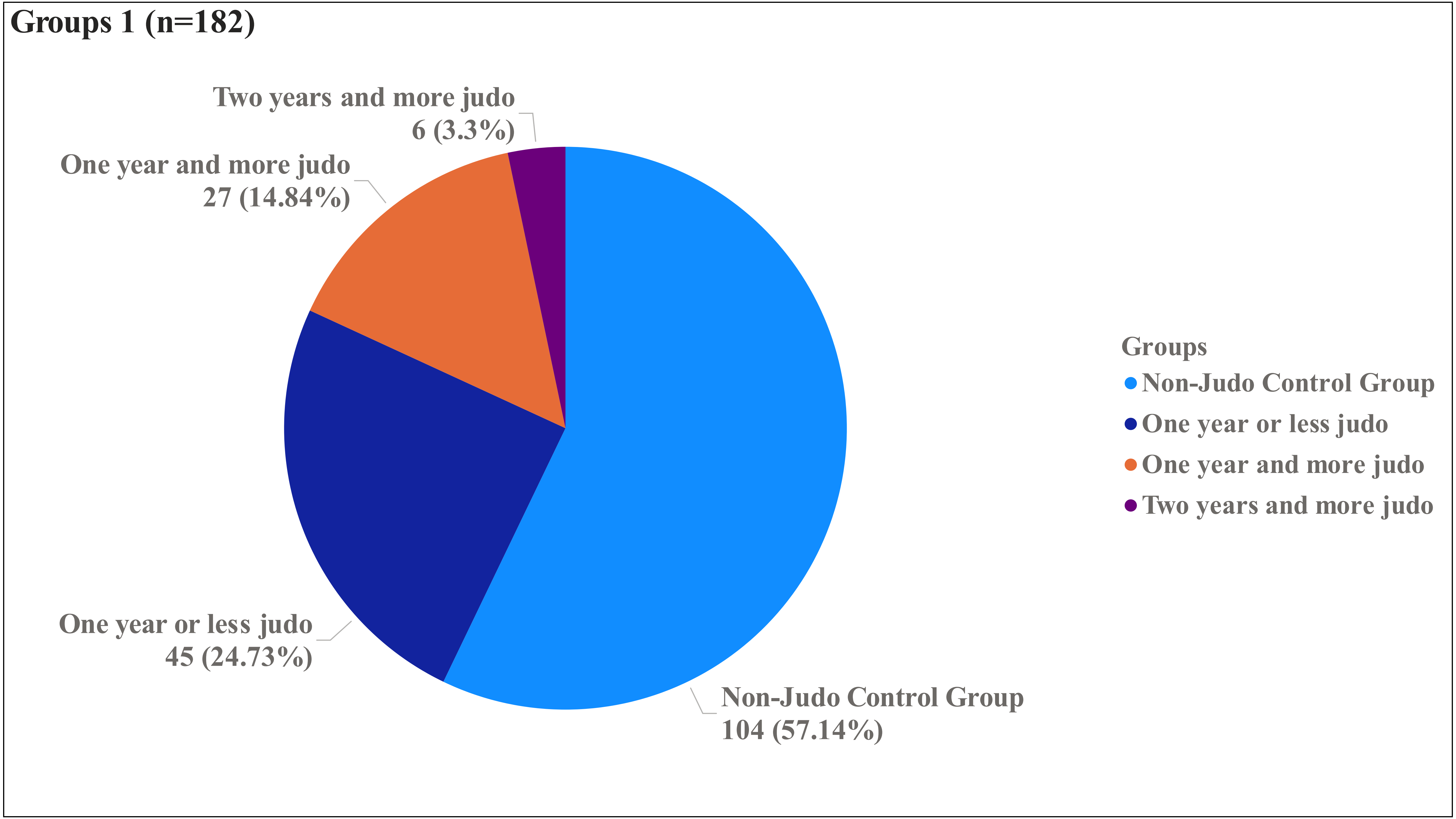
Groups 1 (n=182)
Two years and more judo
6 (3.3%)
One year and more judo
27 (14.84%)
One year or less judo
45 (24.73%)
Non-Judo Control Group
104 (57.14%)
Groups
Non-Judo Control Group
One year or less judo
One year and more judo
Two years and more judo

Countries (n=182)

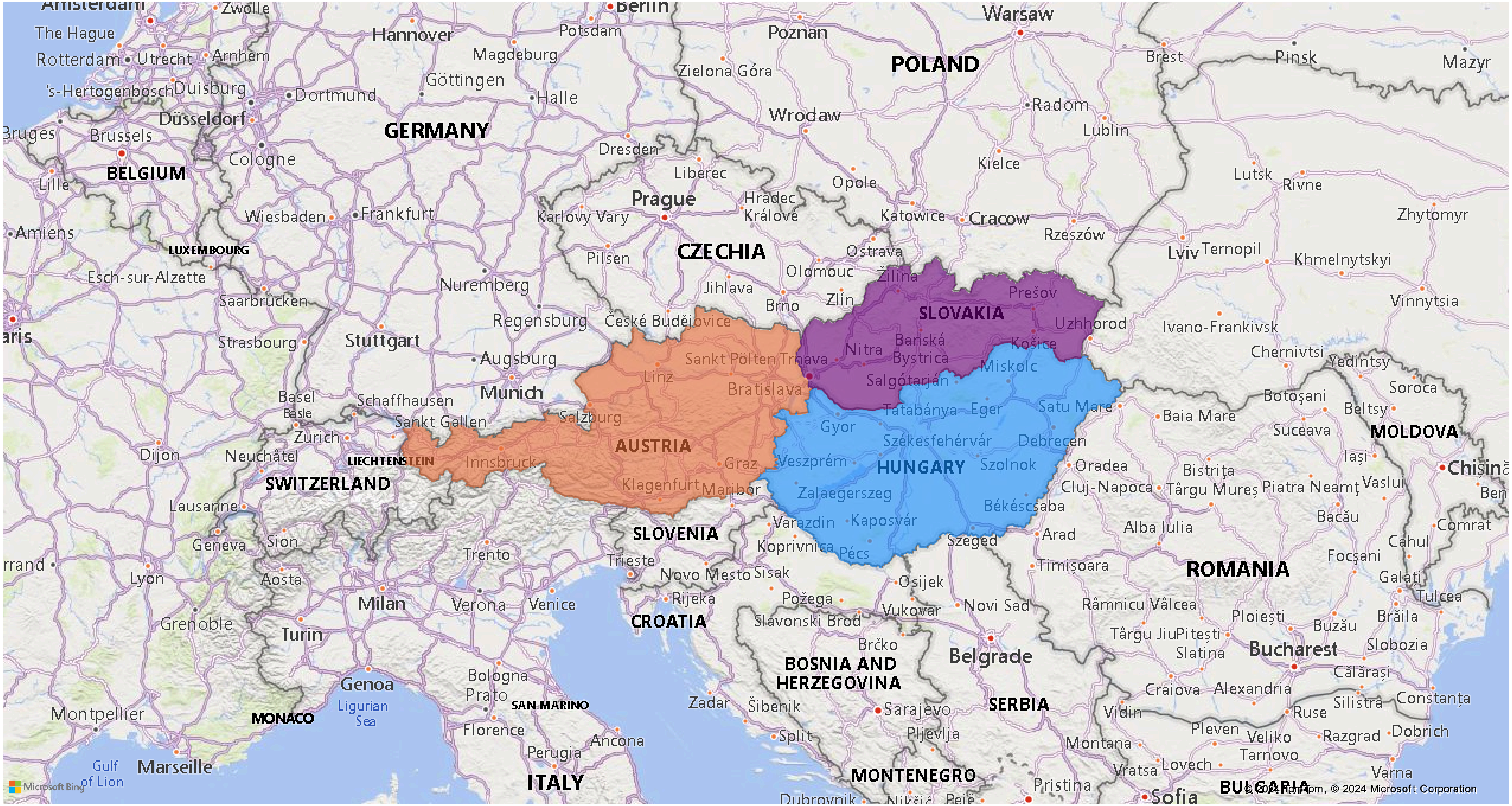

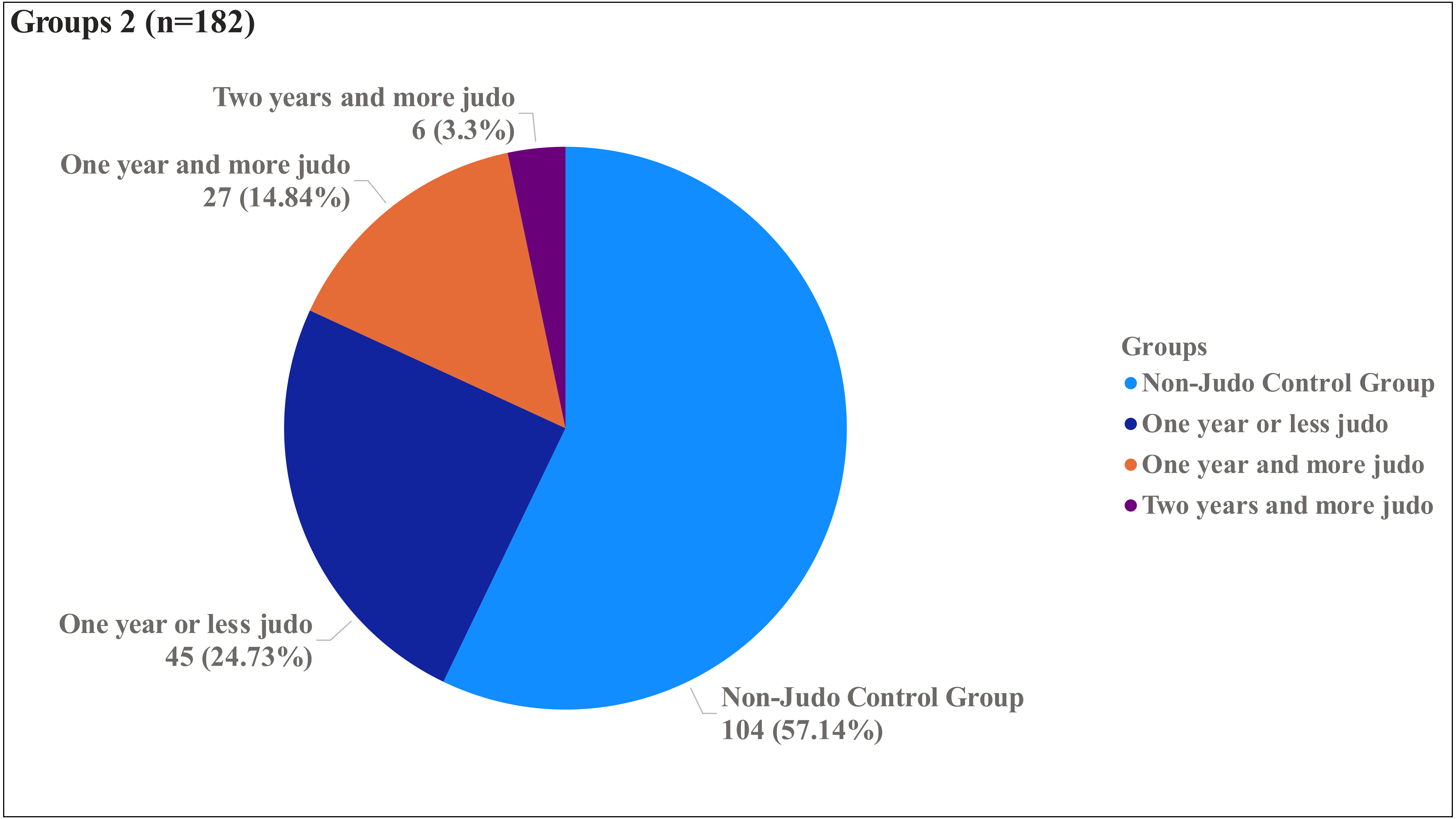
Groups 2 (n=182)
Two years and more judo
6 (3.3%)
One year and more judo
27 (14.84%)
One year or less judo
45 (24.73%)
Non-Judo Control Group
104 (57.14%)
Groups
Non-Judo Control Group
One year or less judo
One year and more judo
Two years and more judo

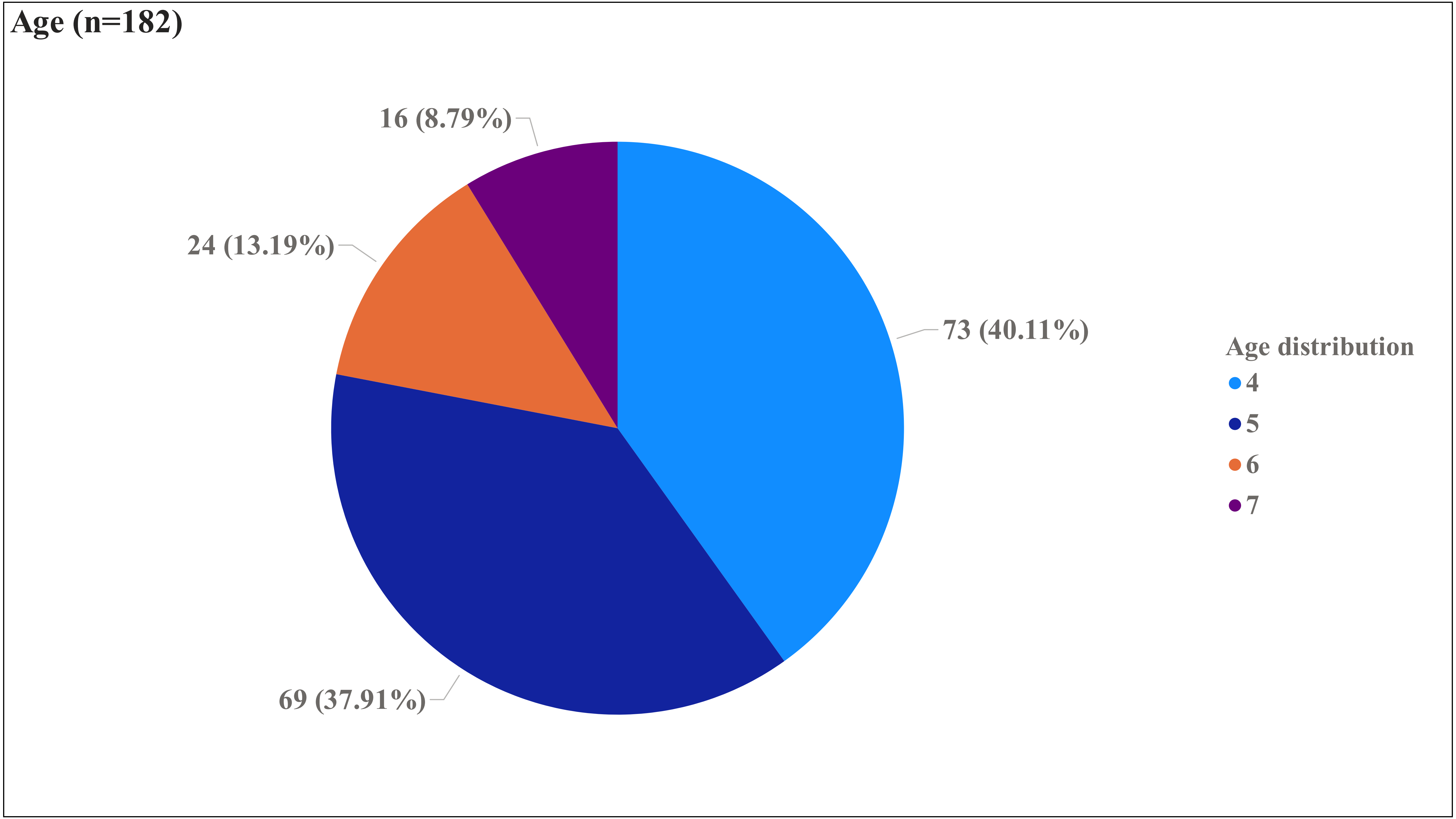
Age (n=182)
16 (8.79%)
24 (13.19%)
73 (40.11%)
69 (37.91%)
Age distribution
4
5
6
7
