# Supplementary material for: Neurodevelopmental benefits of judo training in preschool children: a multinational, mixed methods follow-up study
Source: Front Psychol. 2024 Dec 18;15:1457515. doi: 10.3389/fpsyg.2024.1457515 (PMC11691969; doi:10.3389/fpsyg.2024.1457515)
Supplement: Supplementary file 2 [file Data_Sheet_2.PDF]

Results

Correlation Matrix

		PANESS 1	Neuromotor Test 1	Visual Perceptual Test 1	PSQ Motor Skills	PSQ Process Skills	PSQ Communication	PANESS 2	Neuromotor Test 2	Visual Perceptual Test 2	Sum PSQ
Correlation Matrix											
**PANESS 1**	**Spearman's rho**	—									
	**df**	—									
	**p-value**	—									
**Neuromotor Test 1**	**Spearman's rho**	0.564***	—								
	**df**	78	—								
	**p-value**	<.001	—								
**Visual Perceptual Test 1**	**Spearman's rho**	0.370***	0.174	—							
	**df**	78	78	—							
	**p-value**	<.001	0.122	—							
**PSQ Motor Skills**	**Spearman's rho**	-0.185	-0.146	-0.266*	—						
	**df**	78	78	78	—						
	**p-value**	0.100	0.197	0.017	—						
**PSQ Process Skills**	**Spearman's rho**	-0.050	-0.165	-0.222*	0.811***	—					
	**df**	78	78	78	78	—					
	**p-value**	0.659	0.145	0.047	<.001	—					
**PSQ Communication**	**Spearman's rho**	-0.091	-0.091	-0.167	0.563***	0.651***	—				
	**df**	78	78	78	78	78	—				
	**p-value**	0.424	0.421	0.140	<.001	<.001	—				
**PANESS 2**	**Spearman's rho**	0.897***	0.506***	0.254*	-0.164	-0.031	-0.021	—			
	**df**	78	78	78	78	78	78	—			
	**p-value**	<.001	<.001	0.023	0.146	0.787	0.856	—			
**Neuromotor Test 2**	**Spearman's rho**	0.652***	0.770***	0.239*	-0.264*	-0.208	-0.116	0.718***	—		
	**df**	78	78	78	78	78	78	78	—		
	**p-value**	<.001	<.001	0.032	0.018	0.064	0.304	<.001	—		
**Visual Perceptual Test 2**	**Spearman's rho**	0.499***	0.209	0.765***	-0.199	-0.174	-0.105	0.441***	0.331**	—	
	**df**	78	78	78	78	78	78	78	78	—	
	**p-value**	<.001	0.063	<.001	0.077	0.123	0.356	<.001	0.003	—	
**Sum PSQ**	**Spearman's rho**	-0.100	-0.149	-0.247*	0.874***	0.942***	0.818***	-0.059	-0.212	-0.181	—
	**df**	78	78	78	78	78	78	78	78	78	—
	**p-value**	0.379	0.187	0.027	<.001	<.001	<.001	0.605	0.059	0.109	—

Note. * p < .05, ** p < .01, *** p < .001

References

[1] The jamovi project (2024). *jamovi*. (Version 2.6) [Computer Software]. Retrieved from https://www.jamovi.org.

[2] R Core Team (2024). *R: A Language and environment for statistical computing*. (Version 4.4) [Computer software]. Retrieved from https://cran.r-project.org. (R packages retrieved from CRAN snapshot 2024-08-07).
