# Supplementary material for: Neurodevelopmental benefits of judo training in preschool children: a multinational, mixed methods follow-up study
Source: Front Psychol. 2024 Dec 18;15:1457515. doi: 10.3389/fpsyg.2024.1457515 (PMC11691969; doi:10.3389/fpsyg.2024.1457515)
Supplement: Supplementary file 3 [file Data_Sheet_3.PDF]

Results

Descriptives

	Groups	Gender Distribution	Country	Age	Neuromotor Test 1	Neuromotor Test 2	Visual Perceptual Test 1	Visual Perceptual Test 2	INPP 1	INPP 2	PANESS1	PANESS 2
Descriptives												
**N**	**Non-judo group**	104	104	104	104	104	104	104	104	104	104	104
	**Less than one year judo group**	45	45	45	45	45	45	45	45	45	45	45
	**More than one year judo group**	27	27	27	27	27	27	27	27	27	27	27
	**More than two years judo group**	6	6	6	6	6	6	6	6	6	6	6
**Missing**	**Non-judo group**	0	0	0	0	0	0	0	0	0	0	0
	**Less than one year judo group**	0	0	0	0	0	0	0	0	0	0	0
	**More than one year judo group**	0	0	0	0	0	0	0	0	0	0	0
	**More than two years judo group**	0	0	0	0	0	0	0	0	0	0	0
**Mean**	**Non-judo group**	1.45	1.80	4.56	10.9	9.38	5.23	4.60	18.9	15.5	59.9	53.0
	**Less than one year judo group**	1.29	2.07	4.76	12.6	5.40	4.44	3.22	19.0	9.82	46.7	33.5
	**More than one year judo group**	1.11	1.41	6.15	8.41	4.04	2.56	1.70	11.1	5.74	34.9	21.4
	**More than two years judo group**	1.33	1.50	6.50	7.50	3.50	2.50	0.667	10.0	4.17	29.8	15.3

[a] More than one mode exists, only the first is reported

	Groups	Gender Distribution	Country	Age	Neuromotor Test 1	Neuromotor Test 2	Visual Perceptual Test 1	Visual Perceptual Test 2	INPP 1	INPP 2	PANESS1	PANESS 2
**Std. error mean**	**Non-judo group**	0.0490	0.0617	0.0624	0.650	0.588	0.545	0.531	1.13	1.03	2.49	2.49
	**Less than one year judo group**	0.0683	0.0921	0.111	1.42	0.491	0.785	0.656	2.06	1.11	3.71	2.95
	**More than one year judo group**	0.0616	0.0964	0.166	1.39	0.767	0.593	0.483	1.75	1.08	3.65	3.19
	**More than two years judo group**	0.211	0.224	0.342	2.14	1.69	2.11	0.333	3.76	1.74	6.43	3.07
**Median**	**Non-judo group**	1.00	2.00	4.00	10.0	8.00	4.00	2.00	17.5	14.0	57.0	49.5
	**Less than one year judo group**	1	2	5	10	4	3	2	15	8	48	26
	**More than one year judo group**	1	1	6	5	3	2	1	8	4	30	18
	**More than two years judo group**	1.00	1.50	7.00	6.50	2.50	0.500	0.500	7.00	3.50	25.0	15.0
**Mode**	**Non-judo group**	1.00	2.00	4.00	8.00	8.00	0.00	0.00	8.00[a]	9.00[a]	49.0[a]	53.0
	**Less than one year judo group**	1.00	2.00	5.00	10.0	4.00	0.00	0.00	15.0	8.00	15.0[a]	21.0
	**More than one year judo group**	1.00	1.00	7.00	3.00	2.00	0.00	0.00	3.00[a]	2.00	30.0	9.00
	**More than two years judo group**	1.00	1.00[a]	7.00	2.00[a]	0.00	0.00	0.00	2.00[a]	0.00	16.0[a]	7.00[a]

[a] More than one mode exists, only the first is reported

	Groups	Gender Distribution	Country	Age	Neuromotor Test 1	Neuromotor Test 2	Visual Perceptual Test 1	Visual Perceptual Test 2	INPP 1	INPP 2	PANESS1	PANESS 2
**Sum**	**Non-judo group**	151	187	474	1133	975	544	478	1968	1614	6230	5514
	**Less than one year judo group**	58	93	214	568	243	200	145	854	442	2101	1509
	**More than one year judo group**	30	38	166	227	109	69	46	300	155	943	579
	**More than two years judo group**	8	9	39	45	21	15	4	60	25	179	92
**Standard deviation**	**Non-judo group**	0.500	0.629	0.636	6.63	6.00	5.56	5.41	11.5	10.5	25.4	25.4
	**Less than one year judo group**	0.458	0.618	0.743	9.50	3.29	5.27	4.40	13.8	7.43	24.9	19.8
	**More than one year judo group**	0.320	0.501	0.864	7.22	3.99	3.08	2.51	9.07	5.61	19.0	16.6
	**More than two years judo group**	0.516	0.548	0.837	5.24	4.14	5.17	0.816	9.21	4.26	15.8	7.53
**Variance**	**Non-judo group**	0.250	0.396	0.404	43.9	35.9	30.9	29.3	132	111	647	647
	**Less than one year judo group**	0.210	0.382	0.553	90.3	10.8	27.8	19.4	191	55.1	620	392
	**More than one year judo group**	0.103	0.251	0.746	52.2	15.9	9.49	6.29	82.3	31.5	360	274
	**More than two years judo group**	0.267	0.300	0.700	27.5	17.1	26.7	0.667	84.8	18.2	248	56.7

[a] More than one mode exists, only the first is reported

	Groups	Gender Distribution	Country	Age	Neuromotor Test 1	Neuromotor Test 2	Visual Perceptual Test 1	Visual Perceptual Test 2	INPP 1	INPP 2	PANESS1	PANESS 2
**IQR**	**Non-judo group**	1.00	1.00	1.00	7.50	8.00	9.00	7.00	13.3	11.0	30.0	25.0
	**Less than one year judo group**	1.00	0.00	1.00	9.00	5.00	8.00	4.00	12.0	7.00	34.0	19.0
	**More than one year judo group**	0.00	1.00	1.00	8.50	3.50	2.50	2.00	9.50	5.00	23.5	14.5
	**More than two years judo group**	0.750	1.00	0.750	8.75	4.00	1.00	1.00	7.75	5.50	15.8	9.50
**Range**	**Non-judo group**	1	2	2	45	45	24	24	71	72	114	114
	**Less than one year judo group**	1	2	3	35	13	24	24	71	45	115	91
	**More than one year judo group**	1	1	3	30	14	11	9	38	21	60	59
	**More than two years judo group**	1	1	2	12	11	13	2	25	11	42	20
**Minimum**	**Non-judo group**	1	1	4	0	0	0	0	1	0	14	10
	**Less than one year judo group**	1	1	4	1	0	0	0	1	0	10	10
	**More than one year judo group**	1	1	4	1	0	0	0	3	0	11	5
	**More than two years judo group**	1	1	5	2	0	0	0	2	0	16	7

[a] More than one mode exists, only the first is reported

	Groups	Gender Distribution	Country	Age	Neuromotor Test 1	Neuromotor Test 2	Visual Perceptual Test 1	Visual Perceptual Test 2	INPP 1	INPP 2	PANESS1	PANESS 2
**Maximum**	**Non-judo group**	2	3	6	45	45	24	24	72	72	128	124
	**Less than one year judo group**	2	3	7	36	13	24	24	72	45	125	101
	**More than one year judo group**	2	2	7	31	14	11	9	41	21	71	64
	**More than two years judo group**	2	2	7	14	11	13	2	27	11	58	27
**Skewness**	**Non-judo group**	0.196	0.181	0.703	1.56	2.14	1.12	1.38	1.48	2.12	0.491	0.812
	**Less than one year judo group**	0.964	-0.0376	0.781	1.17	0.385	1.47	2.77	1.69	2.58	0.826	1.68
	**More than one year judo group**	2.62	0.399	-0.690	1.79	1.21	1.81	1.78	2.02	1.30	0.876	1.52
	**More than two years judo group**	0.968	0.00	-1.54	0.300	1.43	2.40	0.857	1.57	0.758	1.38	0.502
**Std. error skewness**	**Non-judo group**	0.237	0.237	0.237	0.237	0.237	0.237	0.237	0.237	0.237	0.237	0.237
	**Less than one year judo group**	0.354	0.354	0.354	0.354	0.354	0.354	0.354	0.354	0.354	0.354	0.354
	**More than one year judo group**	0.448	0.448	0.448	0.448	0.448	0.448	0.448	0.448	0.448	0.448	0.448
	**More than two years judo group**	0.845	0.845	0.845	0.845	0.845	0.845	0.845	0.845	0.845	0.845	0.845

[a] More than one mode exists, only the first is reported

	Groups	Gender Distribution	Country	Age	Neuromotor Test 1	Neuromotor Test 2	Visual Perceptual Test 1	Visual Perceptual Test 2	INPP 1	INPP 2	PANESS1	PANESS 2
**Kurtosis**	**Non-judo group**	-2.00	-0.557	-0.481	5.91	10.8	0.576	1.40	3.99	8.46	-0.0840	0.477
	**Less than one year judo group**	-1.12	-0.241	0.480	0.473	-0.649	2.72	10.6	3.90	10.7	0.879	3.16
	**More than one year judo group**	5.26	-1.99	-0.268	3.30	0.659	2.82	2.63	4.54	1.04	-0.583	1.57
	**More than two years judo group**	-1.87	-3.33	1.43	-2.38	2.07	5.81	-0.300	2.47	-0.206	1.62	-0.426
**Std. error kurtosis**	**Non-judo group**	0.469	0.469	0.469	0.469	0.469	0.469	0.469	0.469	0.469	0.469	0.469
	**Less than one year judo group**	0.695	0.695	0.695	0.695	0.695	0.695	0.695	0.695	0.695	0.695	0.695
	**More than one year judo group**	0.872	0.872	0.872	0.872	0.872	0.872	0.872	0.872	0.872	0.872	0.872
	**More than two years judo group**	1.74	1.74	1.74	1.74	1.74	1.74	1.74	1.74	1.74	1.74	1.74
**Shapiro-Wilk W**	**Non-judo group**	0.633	0.774	0.736	0.905	0.859	0.852	0.816	0.905	0.849	0.971	0.942
	**Less than one year judo group**	0.569	0.773	0.797	0.863	0.955	0.810	0.704	0.861	0.791	0.941	0.844
	**More than one year judo group**	0.368	0.626	0.826	0.795	0.850	0.754	0.722	0.780	0.851	0.859	0.807
	**More than two years judo group**	0.640	0.683	0.701	0.874	0.858	0.571	0.822	0.847	0.917	0.871	0.950

[a] More than one mode exists, only the first is reported

	Groups	Gender Distribution	Country	Age	Neuromotor Test 1	Neuromotor Test 2	Visual Perceptual Test 1	Visual Perceptual Test 2	INPP 1	INPP 2	PANESS1	PANESS 2
**Shapiro-Wilk p**	**Non-judo group**	<.001	<.001	<.001	<.001	<.001	<.001	<.001	<.001	<.001	0.023	<.001
	**Less than one year judo group**	<.001	<.001	<.001	<.001	0.081	<.001	<.001	<.001	<.001	0.023	<.001
	**More than one year judo group**	<.001	<.001	<.001	<.001	0.001	<.001	<.001	<.001	0.001	0.002	<.001
	**More than two years judo group**	0.001	0.004	0.006	0.244	0.182	<.001	0.091	0.150	0.483	0.229	0.739

[a] More than one mode exists, only the first is reported

Frequencies

Frequencies of Gender Distribution

Gender Distribution	Groups	Counts	% of Total	Cumulative %
**Male**	**Non-judo group**	57	31.3%	31.3%
	**Less than one year judo group**	32	17.6%	48.9%
	**More than one year judo group**	24	13.2%	62.1%
	**More than two years judo group**	4	2.2%	64.3%
**Female**	**Non-judo group**	47	25.8%	90.1%
	**Less than one year judo group**	13	7.1%	97.3%
	**More than one year judo group**	3	1.6%	98.9%
	**More than two years judo group**	2	1.1%	100.0%

Frequencies of Country

Country	Groups	Counts	% of Total	Cumulative %
**SK**	**Non-judo group**	33	18.1%	18.1%
	**Less than one year judo group**	7	3.8%	22.0%
	**More than one year judo group**	16	8.8%	30.8%
	**More than two years judo group**	3	1.6%	32.4%
**HU**	**Non-judo group**	59	32.4%	64.8%
	**Less than one year judo group**	28	15.4%	80.2%
	**More than one year judo group**	11	6.0%	86.3%
	**More than two years judo group**	3	1.6%	87.9%
**A**	**Non-judo group**	12	6.6%	94.5%
	**Less than one year judo group**	10	5.5%	100.0%
	**More than one year judo group**	0	0.0%	100.0%
	**More than two years judo group**	0	0.0%	100.0%

Age	Groups	Counts	% of Total	Cumulative %
Frequencies of Age				
**4**	**Non-judo group**	54	29.7%	29.7%
	**Less than one year judo group**	18	9.9%	39.6%
	**More than one year judo group**	1	0.5%	40.1%
	**More than two years judo group**	0	0.0%	40.1%
**5**	**Non-judo group**	42	23.1%	63.2%
	**Less than one year judo group**	21	11.5%	74.7%
	**More than one year judo group**	5	2.7%	77.5%
	**More than two years judo group**	1	0.5%	78.0%
**6**	**Non-judo group**	8	4.4%	82.4%
	**Less than one year judo group**	5	2.7%	85.2%
	**More than one year judo group**	10	5.5%	90.7%
	**More than two years judo group**	1	0.5%	91.2%
**7**	**Non-judo group**	0	0.0%	91.2%
	**Less than one year judo group**	1	0.5%	91.8%
	**More than one year judo group**	11	6.0%	97.8%
	**More than two years judo group**	4	2.2%	100.0%

References

[1] The jamovi project (2024). *jamovi*. (Version 2.6) [Computer Software]. Retrieved from https://www.jamovi.org.

[2] R Core Team (2024). *R: A Language and environment for statistical computing*. (Version 4.4) [Computer software]. Retrieved from https://cran.r-project.org. (R packages retrieved from CRAN snapshot 2024-08-07).
