# Supplementary figures and images for: Neurodevelopmental benefits of judo training in preschool children: a multinational, mixed methods follow-up study
Source: Front Psychol. 2024 Dec 18;15:1457515. doi: 10.3389/fpsyg.2024.1457515 (PMC11691969; doi:10.3389/fpsyg.2024.1457515)

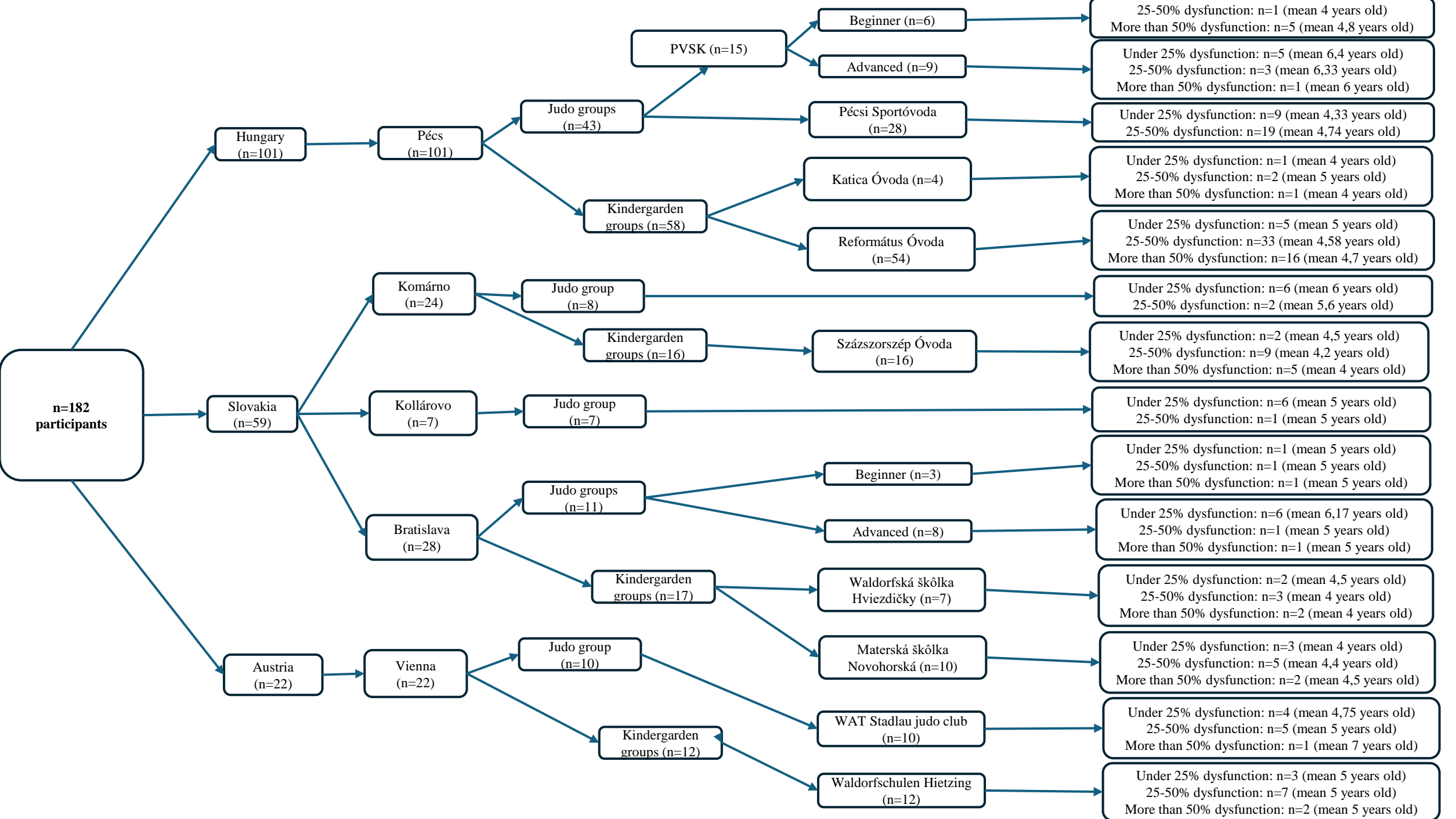

Supplement: Supplementary file 4 [file Data_Sheet_4.PDF]
