# Supplementary material for: Neurodevelopmental benefits of judo training in preschool children: a multinational, mixed methods follow-up study
Source: Front Psychol. 2024 Dec 18;15:1457515. doi: 10.3389/fpsyg.2024.1457515 (PMC11691969; doi:10.3389/fpsyg.2024.1457515)
Supplement: Supplementary file 5 [file Data_Sheet_5.PDF]

Results

Reliability Analysis

	Mean	SD	Cronbach's α	McDonald's ω
Scale Reliability Statistics				
**scale**	0.728	0.502	0.793	0.812

[3]

				If item dropped	
	**Mean**	**SD**	**Item-rest correlation**	**Cronbach's α**	**McDonald's ω**
Item Reliability Statistics					
**RTO 1**	0.0549	0.360	0.233	0.792	0.809
**RTC 1**	0.3077	0.782	0.159	0.793	0.812
**OLSR 1**	1.0275	1.477	0.245	0.792	0.811
**OLSL 1**	1.0385	1.447	0.253	0.791	0.811
**C 1**	1.1484	1.250	0.118	0.797	0.814
**CM1 1**	0.9615	1.228	0.343	0.785	0.805
**CM2 1**	0.4011	1.013	0.478	0.780	0.799
**FTOR 1**	1.3956	1.349	0.308	0.787	0.808
**FTOL 1**	1.4286	1.376	0.282	0.789	0.809
**ATNRR 1**	0.2802	0.843	0.347	0.786	0.802
**ATNRL 1**	0.2527	0.774	0.323	0.787	0.804
**STNRF 1**	0.3462	0.851	0.535	0.779	0.794
**STNRE 1**	1.0549	1.286	0.352	0.785	0.804
**TLRF 1**	0.2088	0.705	0.376	0.786	0.802
**TLRE 1**	0.9341	1.169	0.355	0.785	0.805
**TSFCI 1**	0.4945	1.126	0.405	0.782	0.803
**TSFCR 1**	0.5440	1.219	0.434	0.781	0.802
**TSFS 1**	0.4505	1.110	0.422	0.782	0.803
**TSFX 1**	0.8242	1.472	0.403	0.782	0.804
**TSFT 1**	0.7308	1.317	0.458	0.779	0.801
**TSFUJ 1**	1.5055	1.565	0.267	0.791	0.809
**VL 1**	0.4560	1.215	0.355	0.785	0.805
**HL 1**	0.5055	1.273	0.376	0.784	0.804
**DL 1**	1.1319	1.735	0.380	0.784	0.804

Reliability Analysis

	Mean	SD	Cronbach's α	McDonald's ω
Scale Reliability Statistics				
**scale**	8.66	4.35	0.814	0.862

[3]

				If item dropped	
	Mean	SD	Item-rest correlation	Cronbach's α	McDonald's ω
Item Reliability Statistics					
**4.gaits and stations 1**	16.76	9.75	0.796	0.752	0.812
**5.dysrhytmia 1**	4.63	2.51	0.598	0.807	0.848
**6.overflow movements 1**	4.07	4.22	0.609	0.786	0.846
**7.repetitive movements 1**	3.38	2.45	0.561	0.811	0.854
**8.patterned movements 1**	10.55	6.53	0.772	0.736	0.818
**9.speed 1**	12.54	7.15	0.588	0.784	0.851

Reliability Analysis

	Mean	SD	Cronbach's α	McDonald's ω
Scale Reliability Statistics				
**scale**	0.512	0.412	0.797	0.826

[3]

				If item dropped	
	Mean	SD	Item-rest correlation	Cronbach's α	McDonald's ω
Item Reliability Statistics					
**Second Measurement.RTO 2**	0.0221	0.235	0.450	0.794	0.813
**Second Measurement.RTC 2**	0.2376	0.627	0.234	0.794	0.823
**Second Measurement.OLSR 3**	0.8232	1.203	0.324	0.791	0.822
**Second Measurement.OLSL 4**	0.8232	1.160	0.283	0.793	0.824
**Second Measurement.C 2**	0.4862	0.946	0.232	0.795	0.824
**Second Measurement.CM1 2**	0.5912	1.027	0.268	0.794	0.824
**Second Measurement.CM2 2**	0.0884	0.475	0.318	0.793	0.820
**Second Measurement.FTOR 2**	1.0552	1.084	0.238	0.795	0.825
**Second Measurement.FTOL 2**	1.0663	1.078	0.266	0.794	0.824
**Second Measurement.ATNRR 2**	0.1768	0.598	0.216	0.795	0.822
**Second Measurement.ATNRL 2**	0.1823	0.582	0.194	0.796	0.824
**Second Measurement.STNRF 2**	0.2044	0.565	0.406	0.790	0.816
**Second Measurement.STNRE 2**	0.8287	1.130	0.300	0.792	0.822
**Second Measurement.TLRF 2**	0.1215	0.534	0.269	0.794	0.820
**Second Measurement.TLRE 2**	0.7182	1.029	0.336	0.790	0.821
**Second Measurement.TSFCI 2**	0.3260	0.924	0.504	0.782	0.813
**Second Measurement.TSFCR 2**	0.3591	0.977	0.464	0.783	0.816
**Second Measurement.TSFS 2**	0.3260	0.942	0.560	0.779	0.812
**Second Measurement.TSFX 2**	0.5912	1.277	0.451	0.783	0.817
**Second Measurement.TSFT 2**	0.6906	1.335	0.482	0.781	0.816
**Second Measurement.TSFUJ 2**	1.3867	1.489	0.345	0.792	0.822
**Second Measurement.VL 2**	0.1823	0.734	0.507	0.784	0.811
**Second Measurement.HL 2**	0.1934	0.746	0.477	0.785	0.812
**Second Measurement.DL 2**	0.8122	1.527	0.365	0.791	0.820

Reliability Analysis

	Mean	SD	Cronbach's α	McDonald's ω
Scale Reliability Statistics				
**scale**	7.05	4.34	0.823	0.875

[3]

	Mean	SD	Item-rest correlation	If item dropped Cronbach's α	McDonald's ω
Item Reliability Statistics					
**Second Measurement.4.gaits and stations 2**	13.09	9.42	0.828	0.753	0.826
**Second Measurement.5.dysrhytmia 2**	3.73	2.40	0.610	0.818	0.863
**Second Measurement.6.overflow movements 2**	3.81	4.10	0.633	0.796	0.861
**Second Measurement.7.repetitive movements 2**	3.13	2.42	0.610	0.818	0.864
**Second Measurement.8.patterned movements 2**	8.28	6.19	0.776	0.751	0.837
**Second Measurement.9.speed 2**	10.23	7.60	0.594	0.801	0.867

References

[1] The jamovi project (2024). *jamovi*. (Version 2.6) [Computer Software]. Retrieved from https://www.jamovi.org.

[2] R Core Team (2024). *R: A Language and environment for statistical computing*. (Version 4.4) [Computer software]. Retrieved from https://cran.r-project.org. (R packages retrieved from CRAN snapshot 2024-08-07).

[3] Revelle, W. (2023). *psych: Procedures for Psychological, Psychometric, and Personality Research*. [R package]. Retrieved from https://cran.r-project.org/package=psych.
