# Supplementary material for: Neurodevelopmental benefits of judo training in preschool children: a multinational, mixed methods follow-up study
Source: Front Psychol. 2024 Dec 18;15:1457515. doi: 10.3389/fpsyg.2024.1457515 (PMC11691969; doi:10.3389/fpsyg.2024.1457515)
Supplement: Supplementary file 6 [file Data_Sheet_6.PDF]

Results

Reliability Analysis

	Mean	SD	Cronbach's α	McDonald's ω
Scale Reliability Statistics				
**scale**	5.19	0.630	0.954	0.959

[3]

	Mean	SD	Item-rest correlation	If item dropped	
				Cronbach's α	McDonald's ω
Item Reliability Statistics					
**Sits stable, stands straight maintains trunk control and balance, does not fall**	5.66	0.835	0.537	0.953	0.958
**Walks and runs easily, climbs stairs, skips, swings himself**	5.74	0.718	0.615	0.953	0.958
**Efficiently uses small objects, without tremor, doesn't drop or spills liquid**	5.49	0.915	0.708	0.952	0.957
**Uses preferred hand consistently**	5.51	0.935	0.569	0.953	0.958
**Lifts, pushes, pulls heavy objects, has strength**	5.62	0.832	0.587	0.953	0.958
**Regulates or grades force appropriate to task and objects**	5.44	0.826	0.683	0.952	0.957
**Grasp efficiently, does not drop**	5.57	0.754	0.696	0.952	0.957
**Persists during physical tasks without fatigue**	5.15	0.950	0.563	0.953	0.958
**Maintains a consistent and effective rate or tempo of performance throughout the entire task**	4.88	1.043	0.613	0.953	0.958
**Calm—he is not hyperactive nor hypo active**	4.67	1.446	0.379	0.956	0.959
**In a task or activity chosen by the child-persists and completes the task, maintains focused attention, is not distracted**	5.19	0.973	0.566	0.953	0.958
**In a task chosen by others—persists and completes the task, maintains focused attention, is not distracted**	4.42	1.116	0.626	0.953	0.958
**Selects and uses appropriate tools and materials for a task or activity**	5.50	0.805	0.840	0.951	0.956
**Seeks for help, asks when uncertain, doesn't ask if not necessary**	5.12	0.929	0.621	0.953	0.958
**Begins an activity easily and continues in sequentially and continuously**	4.84	0.992	0.762	0.952	0.957
**Performs activity in the right sequence order**	5.11	0.994	0.734	0.952	0.957
**Completes activity or a task within the time guidelines (proc7)**	4.71	1.073	0.651	0.952	0.958
**Looks for and locates objects in a logical manner (proc8)**	5.39	0.844	0.735	0.952	0.957
**Uses tools and materials in an orderly fashion**	5.39	0.855	0.708	0.952	0.957
**Gathers toys, put objects back in the right place**	4.92	1.108	0.516	0.953	0.959
**Modifies his movement and avoids knocking over or bumping into objects**	5.32	1.194	0.687	0.952	0.958
**Tries to find solutions, shifts his action in accordance to circumstances**	5.23	0.853	0.795	0.952	0.957
**Learns from his mistakes, willing to receive help**	4.95	1.013	0.657	0.952	0.958
**Adapts easily to changes and transitions**	4.66	1.213	0.453	0.954	0.959
**Keeps the right distance from people doesn't touch them**	5.12	1.088	0.268	0.955	0.960
**Initiates eye contact**	5.30	1.039	0.315	0.955	0.960
**Uses common gesture and expressions for communication**	5.56	0.849	0.779	0.952	0.957

	Mean	SD	Item-rest correlation	If item dropped	
				Cronbach's α	McDonald's ω
**Produce clear understandable speech, has no articulation disruptions**	5.15	1.272	0.629	0.953	0.958
**Uses appropriate volume and inflection in speech according to situation**	5.16	0.982	0.611	0.953	0.958
**Expresses himself easily, speaks properly and directly expresses desires and requests, is able to give descriptions**	5.29	1.055	0.718	0.952	0.957
**Conforms, respects rules at home and school**	4.93	1.016	0.634	0.953	0.958
**Acts politely, asks for permission, awaits his turn**	5.00	1.046	0.597	0.953	0.958
**Negotiates, resolves conflicts, collaborates with peers, is not obnoxious**	5.16	1.047	0.576	0.953	0.958
**Respects, relates and helps others**	5.26	0.945	0.585	0.953	0.958

References

[1] The jamovi project (2024). *jamovi*. (Version 2.6) [Computer Software]. Retrieved from https://www.jamovi.org.

[2] R Core Team (2024). *R: A Language and environment for statistical computing*. (Version 4.4) [Computer software]. Retrieved from https://cran.r-project.org. (R packages retrieved from CRAN snapshot 2024-08-07).

[3] Revelle, W. (2023). *psych: Procedures for Psychological, Psychometric, and Personality Research*. [R package]. Retrieved from https://cran.r-project.org/package=psych.
